# Incidental covariation learning leading to strategy change

**DOI:** 10.1371/journal.pone.0210597

**Published:** 2019-01-24

**Authors:** Robert Gaschler, Nicolas W. Schuck, Carlo Reverberi, Peter A. Frensch, Dorit Wenke

**Affiliations:** 1 Department of Psychology, FernUniversität in Hagen, Hagen, Germany; 2 Research Cluster Image Knowledge Gestaltung at Humboldt-Universität Berlin, Berlin, Germany; 3 Max Planck Research Group NeuroCode, Max Planck Institute for Human Development, Berlin, Germany; 4 Department of Psychology, Università Milano–Bicocca, Milano and Milan Center for Neuroscience, Milano, Italy; 5 Department of Psychology, Humboldt-Universität Berlin, Berlin,Germany; 6 Private University of Applied Sciences, Göttingen, Germany; Fordham University, UNITED STATES

## Abstract

As they approach a traffic light, drivers and pedestrians monitor the color (instructed stimulus feature) and/or the position of the signal (covarying stimulus feature) for response selection. Many studies have pointed out that instructions can effectively determine the stimulus features used for response selection in a task. This leaves open whether and how practice with a correlating alternative stimulus feature can lead to a strategy change from an instructed to a learned variant of performing the task. To address this question, we instructed participants to respond to the position of a stimulus within a reference frame, at the same time, during task performance, an unmentioned second stimulus feature, the color, covaried with stimulus position and allowed the use of an alternative response strategy. To assess the impact of the non-instructed stimulus feature of color on response selection throughout practice, the spatial position of the stimulus was ambiguous on some trials. Group average increases in color usage were based on a mixture of (1) participants who, despite extended practice on the covariation, exclusively relied on the instructed stimulus feature and (2) those who abruptly started to rely heavily on stimulus color to select responses in ambiguous trials. When the instructed and uninstructed feature predicted different actions, choices were still biased by the uninstructed color feature, albeit more weakly. A second experiment showed that the influence of color generalized across frequently and infrequently presented combinations of position and color. Strategy changes were accompanied by awareness in both experiments. The results suggest that incidental covariation learning can trigger spontaneous voluntary strategy change involving a re-configuration of the instructed task set.

## Introduction

Instructions can determine how we handle a task [1–3]. Yet, the literature on skill acquisition shows that practice improves task performance [4–7]. Both factors–learning [8, 9] as well as instructions [1, 3]–are thought to affect attentional control during task execution, which then causes the processing of task-relevant information to be prioritized over the processing of information that has proven to be, or was instructed to be, less relevant (cf. Miller and Cohen [10]). Research by Doll and colleagues as well as Schmidt and De Houwer has shown that the concurrent influence of learning and instructions on attentional control can lead to interference; for example when instructions hinder the learning of contingencies in the task material [11, 12]. While Dreisbach and Haider have shown that instructions can shield the processing of relevant information from being influenced by irrelevant information [13, 14], this, according to Tsushima and colleagues, does not rule out learning about an irrelevant feature that provides an alternative strategy for performing a task [15].

In many everyday tasks, there is no cognitive conflict [16] between instruction-based task processing and learning-based task processing. Instead, we often react to one feature, while a different one would lead to the same result. For instance, when approaching a traffic light, according to instructions, we should base our actions on color. However, the position of the light would allow for stimulus-identification and response selection as well, because traffic light color and position are perfectly correlated. Thus, with practice we might incidentally acquire the knowledge to instead (or in addition) select the response based on the *position* of the light (for early work on the traffic light example see Overton and Brown [17]). More generally, this research suggests that changes in cognitive control can also be caused by learning about the task structure even in the absence of cognitive conflict.

The current work examines how covariation learning can lead to strategy change in the absence of cognitive conflict and whether this impedes participants from detecting cognitive conflict once it occurs (cf. [18]). While the position of the colors in traffic lights should not be changed due to safety concerns, such a test is informative in our laboratory setup: Would (some) participants base their responses on the formerly covarying feature of color rather than the instructed feature, when the covariation is violated? Changing from position- to color-based response selection might either entail continued checking of the instructed stimulus feature or lead to neglect of conflict between learning and instruction. In the latter case the (formerly) covarying feature would even be used when it no longer leads to a response in line with what instruction-based processing would have yielded. In a setup similar to the current work, Schuck and colleagues [19] instructed participants to react to stimulus position and tracked via behavioral measures and fMRI pattern classification when and how participants came to use the covarying feature stimulus color. While the instructed stimulus feature position as well as the irrelevant feature of color could both be decoded from visual processing areas, only stimulus position could be decoded from prefrontal areas at the beginning of the experiment. Yet, with practice, the medial prefrontal cortex came to contain color information for participants if and right before they started to use it. The change from selecting responses based on position (as instructed) to responding based on color could be predicted by tracking the information represented in the medial prefrontal cortex. This representation changed before the behavioral change. This finding suggests that the medial prefrontal cortex might be involved in vicariously simulating the use of the alternative stimulus feature before overtly using it. Furthermore, participants using the covariation for selecting responses were aware of the covariation.

While the above study can be taken to suggest that covariation learning can lead to a strategy change that involves a changing representation of the task (from a react-to-position task to reacting to color–instead of or in addition to position), there were no trials in which the color-position covariation was violated. Thus, it was not investigated whether participants might apply color-based response selection even when it leads to a different response than what instruction-based processing would yield. Therefore, more observational evidence and experimental manipulations are needed to further test this perspective. The observations by Schuck et al. showed two features attributed to voluntary strategy change by Haider and colleagues: Sudden onsets of change in performance [20] paired with awareness about the regularity in the task material [21]. We therefore scrutinized the central assumptions of different theories of learning-based strategy change by manipulating the composition of the practice material.

The theories describing learning-based strategy change can be broadly categorized into two groups. According to one view, the shift from instruction-based processing to a shortcut is a direct and inevitable consequence of task practice. It is tied to how often the specific material has been practiced [5, 6, 22]. In tasks such as mental arithmetic, participants will show strategy changes one at a time, first with the often-practiced task material and later with less often practiced material. A voluntary decision is not involved. For instance, according to Logan [5, 6], automatic encoding and the retrieval of memory traces lead to a transition from a slow, calculation-based strategy to faster, memory-based responding as a mandatory consequence of task processing. With every trial, the number of memory traces containing past stimuli and responses increases. So does the probability that memory retrieval rather than calculation will determine the response to a problem. The more traces race in parallel for retrieval from memory, the larger the chance that the fastest of these will trigger the response before calculation is completed.

The alternative view assumes that a voluntary decision is involved. For instance, some of the participants in the previously mentioned study by Schuck and colleagues [19] could report the covariation of color and position after the experiment, but had not used it for response selection. While automatic (i.e., involuntary and often implicit) learning provides knowledge about the regularities in the task material, it does not necessarily lead to strategy change. First, participants will generate knowledge about the exploitable regularities in the task material. Then they will decide to use this knowledge, reducing cognitive effort by switching to a more efficient task strategy [21–26]. Participants switching to the more efficient strategy were able to report on the underlying regularity in the task material they had discovered, when the experiment was interrupted for interviewing right after a spontaneous strategy change had occurred [21]. According to Gaschler and colleagues as well as to Haider and Frensch, the decision to use this knowledge can lead to a strategy change for often-practiced, less practiced, and even novel variants of the task material alike [27, 28]. In line with this, generalization of a strategy to novel or less-practiced stimuli can be seen as an indicator of voluntary strategy change [29–32]. If implementing the new strategy on the psychomotor level is straightforward (i.e., possible without practice), then, according to Haider and colleagues, the decision for strategy change can be accompanied by an abrupt change in performance [21, 28].

It is important to note that in past work on strategy change, participants learned to skip parts of the stimulus processing they had been instructed to perform. There was a change in the *extent* to which the instructed stimulus feature was being processed (i.e., skipping to check aspects of the stimulus in a verification task, [28]). Yet, there was no alternative stimulus feature, which, despite not being mentioned in the instructions, could be used as an alternative source of response selection after learning [19]. It is also worth noting that these views on strategy change are not necessarily mutually exclusive. Prior work by Woltz and colleagues as well as Doane and colleagues has shown that at least some types of strategy change can take place without a top-down decision and outside the awareness of participants [18, 33].

While the Schuck et al study [19] yielded some insights in how a covarying stimulus feature not mentioned in the instructions can lead to strategy change, theoretical as well as methodological issues remain to be resolved. In [19], the covarying color could be decoded from prefrontal areas of the brain once participants had enough practice to execute a strategy change. Yet, the design of the study did not allow for testing for generalization across colors. While participants could have exclusively relied on the specific associations between color and position, it is also possible that knowledge about one color-position association might have played a role in establishing the response mapping for the other color as well. Past work suggests the effectiveness of the mere notion that there might be *some* shortcut to be found and used. For instance, Gaschler and colleagues [34] found that participants who worked on a task in which they were able to discover that a strategy change is feasible were more likely to discover and use a shortcut in the next task–despite that the two tasks shared neither stimuli nor responses and the shortcut was rather different (stopping to attend to an aspect of the visual stimulus vs. substituting stimulus-based processing by memory-retrieval). The only shared feature had been that *a* shortcut was possible.

**Fig 1:**
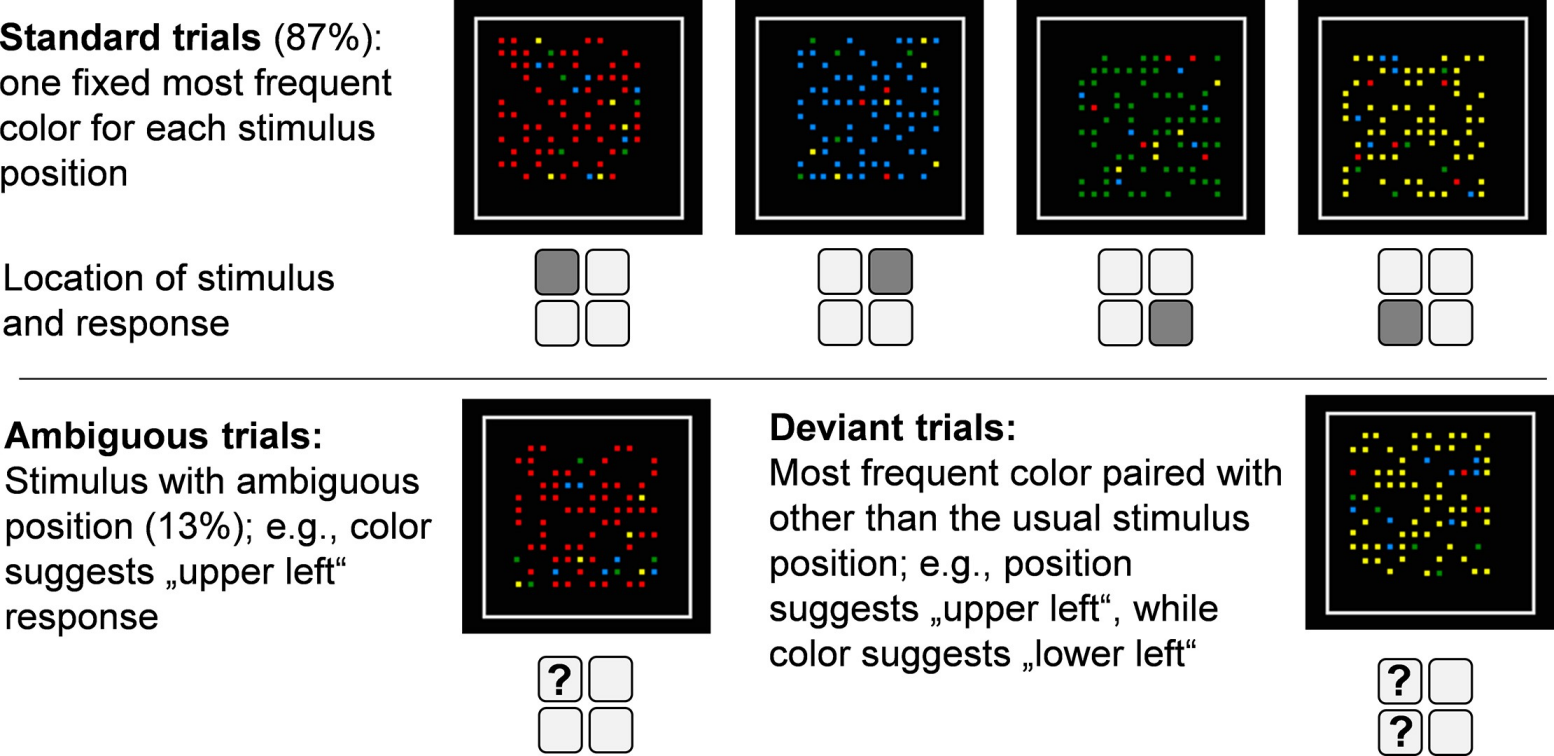
Examples of stimuli. In standard and deviant trials the instructed feature position is identifiable and can be used to select a response (mapping in Exp. 1: press the button corresponding to the corner of the reference frame the cloud of small squares is closest to). While in standard trials the most frequent color covaries with position, this coupling is violated in deviant trials. In ambiguous trials, the instructed feature position cannot be identified and used to select a response; as a result, only the color-position covariation present in the standard trials can influence response selection.

Furthermore, it remains open whether the test trials used to behaviorally assess color usage in [19] induced the very phenomenon they were assessing. Ambiguous stimuli (see Fig 1) were used to obtain a behavioral indicator of strategy change. In these trials, the stimulus position (the instructed stimulus feature) could not be discriminated. This allowed the assessment of whether participants would choose a response based on the covariation between stimulus position and color. However, according to Rünger and Frensch, it is conceivable that special control- or search processes are triggered by probing a participant with a stimulus that cannot be easily discriminated based on the instructed stimulus feature [35]. Therefore, it is necessary to test whether this measure is reactive.

Our setup (Fig 1) allowed us to assess whether incidental covariation learning during task processing occurred and led to a change in the way the task was performed. On each trial, participants were presented with an array of small colored squares, which were located within a square reference frame. In most of the trials, the array was placed in closer proximity to one of the corners of the reference frame. In Experiment 1, the response keys were arranged in a square such that each stimulus position was mapped onto a spatially corresponding response-key layout with the four keys: If the array was closest to the upper left corner, participants were to press the upper left key, etc. The small squares that made up the array had one of four colors, but on any given trial one of the colors was most frequent. There was a fixed relation between array color and array location. For instance, whenever the array was located in the upper left corner it consisted mostly of green elements. Thus, participants could, in principle, use stimulus position and/or stimulus color for response selection. Participants were not informed about this contingency, but could learn and apply it spontaneously. The results obtained in [19] suggest that covariation learning leads to strategy change accompanied by awareness about the regularity of the task material.

In order to test whether ambiguous stimuli are producing the very phenomenon they are meant to measure, in Experiment 1 we manipulated the time point at which the ambiguous trials were introduced during the experiment. Different from the *ambiguous-throughout condition*, participants in the *ambiguous-after-learning condition* and the *no-ambiguous condition* received ambiguous trials either only late in practice or not at all. Comparing between these groups allowed us to assess any potential effect the mere presence of ambiguous trials could have on color usage. A second type of test trial was only introduced at the end of the experiment and involved uncommon pairings of stimulus color and stimulus position. In these *deviant* trials, the stimulus position signaled a different response than the stimulus color. This allowed us to test whether participants would follow the covarying feature of color *instead* of the instructed feature in response selection–or whether processing of the irrelevant feature would at least delay the response.

Experiment 2 aimed at exploring the processes underlying strategy change due to covariation learning by manipulating the frequency with which specific color-position pairs were presented. It aimed at testing whether participants started to use each color individually or would show generalization across color-position pairs and a consistent shift in response selection towards incorporating this feature. Participants might (not only) learn that a specific stimulus position is paired with a specific color, but also acquire the more general regularity–that positions have a fixed assignment to colors. This implies that strategy change might generalize from frequent to infrequent color-position pairs.

## Experiment 1

### Methods

#### Participants

Sixty six volunteers (48 female, mean age 24.8 years, *SD* = 6.6 years, one left-handed) participated in the study in return for course credit or €7. The sample size was sufficient to detect small effects in interactions of between- and within-subjects factors (i.e., whether type of stimulus played out differently among the three groups) at a power of .95, according to Faul et al. [36].

#### Task and procedure

We obtained approval from the ethics committee of the department of psychology of Humboldt-Universität, Berlin. In both experiments participants were tested after obtaining informed consent.

Participants were instructed to respond manually to the location of an array of colored squares within a square reference frame (Fig 1). The reference frame measured 4.3 by 4.3cm (100 pixels) on a 17inch CRT screen running at 800 by 600 pixels resolution. Color squares that made up the array measured 3 by 3 pixels. An array was defined as a grid of 12 by 12 squares, half of which were colored and half of which were empty. Filled vs. empty squares were evenly distributed across the quadrants of the array, resulting in the overall impression of a larger square. The spatial position of an array was defined by a shift of the array relative to the reference frame. Arrays were shifted by 6 pixels in the x and y dimensions relative to the center of the reference frame. We instructed a simple 1:1 mapping of stimulus locations (upper left, upper right, lower right, lower left corner) to keys on the number pad of the keyboard. Participants used left and right index and middle fingers on the respective corner-keys of the number pad of a German keyboard marked by white stickers. They quickly responded to array position. Participants were instructed to guess on trials in which they could not determine the location of the array. While we mentioned that the arrays were colored, no further information concerning color was given. Participants were not told that the colors of the squares would be informative with respect to the required response in some parts of the experiment.

Each trial started with the presentation of an array of colored squares within a white reference frame on a black background. The small squares that made up the array could be green, blue, red and brown at equal luminance. Arrays consisted of small squares of all colors, but one color was dominant on each trial; the dominant color was used for 56 (77.78%) of the 72 colored squares while the other three colors were used to fill the remaining 16 squares (22.22%). The array disappeared with a participant’s response or 500ms after trial onset. Although the stimulus sometimes disappeared before the participant had made a choice, responses could be made up to 2500ms after trial onset. The next trial started 100ms after the response. Participants completed a total of 3072 trials separated into 24 blocks of 128 trials each within 50 to 60 minutes. They were instructed to respond as accurately and quickly as possible and received feedback after each block concerning average reaction time (RT) and error rate (based on standard trials only). Errors in standard trials were followed by a 100ms tone of 200Hz.

After two blocks in which the dominant color of the arrays was not related to stimulus location on standard trials, the two stimulus features became deterministically coupled. We started with random blocks in order to have a baseline even in case of a very quick onset of color usage. Balanced across participants each of the four stimulus positions was paired with one specific dominant color. For instance, an array of squares could consist mostly of green dots whenever it appeared in the upper left corner. Only two varieties of test trials–spatially ambiguous trials and deviant trials—differed from this typically fixed coupling.

(1) To continuously assess color usage over the course of practice, we interspersed spatially ambiguous test trials (16 per block = 12.5%) from Block 1 to Block 21 in the ambiguous-throughout condition, and from Block 13 to Block 21 in the ambiguous-after-learning condition. In the ambiguous trials, the array was positioned exactly in the middle of the reference frame rather than shifted towards one of the four corners. In order to avoid deliberation processes, participants were instructed in advance that on some trials array position would be difficult to identify, and that they should just go with guessing on such trials.

(2) In the last three blocks of the experiment, deviant trials (12.5%) served as test trials instead of ambiguous trials. In the deviant trials, array position and dominant color were paired in a different way than experienced in standard trials (during learning). As deviant trials allowed for unambiguous identification of the instructed stimulus attribute (i.e., array position), it was possible to explore whether the participants would respond according to the instructed stimulus location (e.g., upper left key when the stimulus is positioned in the upper left corner) or according to the formally irrelevant but usually correlated stimulus feature of color when both dimensions signaled a different response. For instance, the lower right key might be pressed, because usually blue stimuli occur in the lower right corner and lead to a response on the lower right key. We also analyzed the RT slowing of “correct” responses to stimulus position in deviant as compared to standard trials as a measure of interference from the irrelevant feature of color. Although one could argue that deviant trials provide a more comprehensive measure for the influence of color processing than ambiguous trials, introducing deviant trials only during the last few blocks had the advantage of not violating/weakening the color-position covariation during the initial learning phase (cf. detrimental effects of random events during learning in Rünger and Frensch [35]).

No “error” feedback was given on either ambiguous or deviant test trials (i.e., every response counted as correct). This was done in order to avoid biasing learning and/or use of the irrelevant color feature.

### Results

#### Raw data

Raw data of both experiments are available at: https://osf.io/z53yj/

#### Screening of the data

We excluded six participants with error rates > = 25% in standard trials, leaving 20 participants in the ambiguous-throughout condition, 22 in the ambiguous-after-learning condition and 18 in the no-ambiguous condition. The average error rate in standard trials for the remaining participants were 11.16%, 9.8%, and 9.23% for the three groups (*F*<1).

#### Overview of the analyses

We first report data showing how the participants started to select responses according to the covarying stimulus feature of color as they gained exposure. To this end, we focus on color usage in ambiguous test trials during the learning phase. We then explore the extent to which color was used when pitted against the instructed feature position, and whether responses in line with the stimulus position were slowed in deviant trials.

#### Average rate of color usage in ambiguous trials

**Fig 2:**
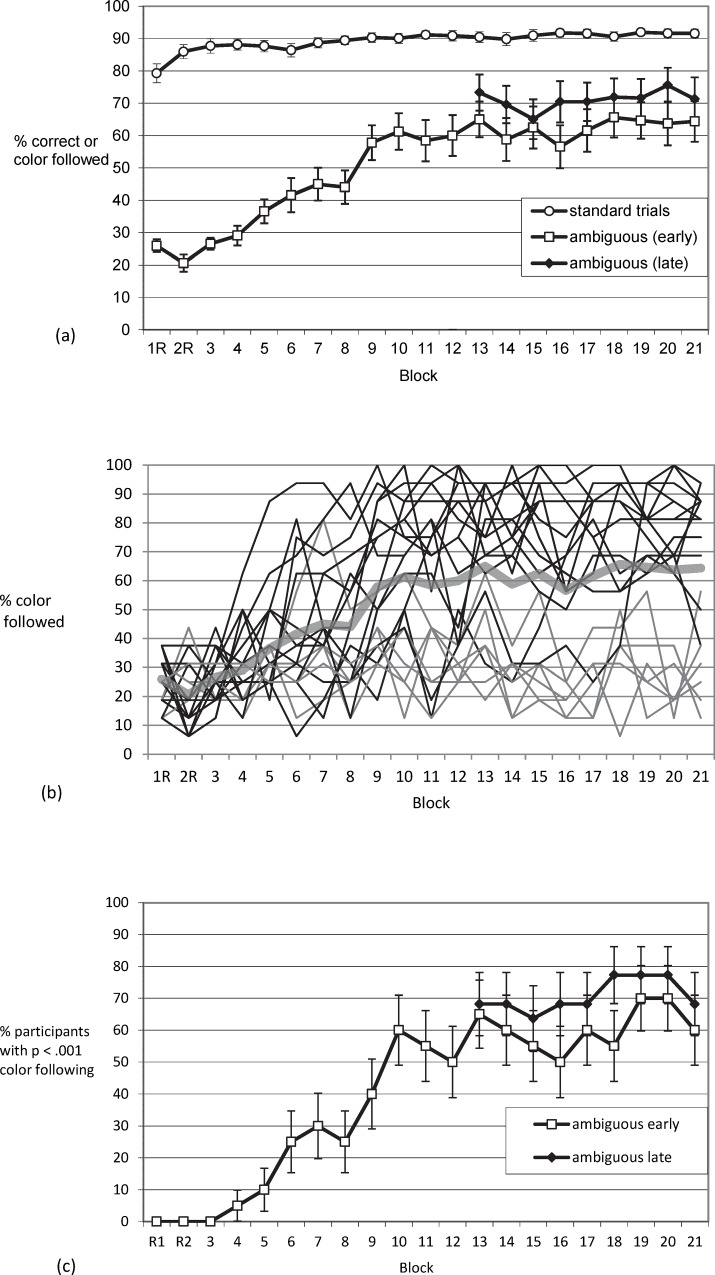
Exp. 1: Color usage in ambiguous trials. Panel (a) shows that the average proportion of ambiguous trials that elicit a response in line with the color-position covariation increases with practice in the ambiguous-throughout condition. The ambiguous-after-learning condition sets in with a similar rate of color usage in ambiguous trials. Panel (b) shows that continuous increases found when averaging across participants are not representative for the dynamics in the individual participant: Some participants show strong increases in color usage (some early in practice, others late in practice), while others never show strong color usage. In line with this, Panel (c) suggests that the average increase of color usage with practice is a mixture phenomenon–being mainly driven by the increasing proportion of participants who at a certain point in practice already show strong color usage. Error bars depict the between subjects standard error of the mean.

We first tested whether participants would, with practice, base responses on the non-instructed feature, color. Ambiguous trials allowed us to track the increase of color usage across the blocks of practice in the ambiguous-throughout condition. As shown in Fig 2A, color usage in ambiguous trials increased across blocks of practice from chance level (25%) to a group average of over 50%. Thus, when the instructed feature (stimulus position) could not be used to select a response, participants increasingly relied on the correlated color feature. For instance, with a centrally presented array of predominantly blue dots, participants tended to press the key that was associated with upper left (predominantly blue) stimuli on standard trials.

For the ambiguous-throughout condition, we analyzed the impact of practice on the proportion of ambiguous trials which were responded to in line with the corner-color covariation with a one factorial repeated measures ANOVA (Block 3 to 21 –as in Block 1 and 2 color was assigned randomly). We found a main effect of practice, *F*(4.98, 94.59) = 11.19, *MSE* = 1029.56, *p* < .001, *η*_*p*_^*2*^ = .371, Greenhouse-Geisser-corrected.

In order to test whether the existence of ambiguous trials from the start influenced color usage, over and above measuring learning-based use of color, we compared the ambiguous-throughout condition and the ambiguous-after-learning condition with respect to color usage in ambiguous trials. If ambiguous trials not only assessed but also caused the use of stimulus color, then the group that experienced ambiguous trials later in the experiment should show less color use. No indication for such a methodological problem was present. Groups did not differ with regard to color use once they both experienced ambiguous trials. The mixed ANOVA across the last nine blocks of practice (where both the ambiguous-throughout and the ambiguous-after-learning groups were presented with the same number of ambiguous trials) neither led to a significant main effect of group (*F* = 1.21), nor a main effect of block (*F* = 1.33), nor an interaction (*F* < 1).

#### Rate of participants using color on ambiguous trials

According to Haider and Frensch, voluntary strategy changes are characterized by all-or-none changes in performance [28]. Thus, the steady group average increase in the rate of color usage (Fig 2A) might be based on different participants showing a strategy change at different points in time, with some participants showing no change at all, rather than a steady increase in all participants (see also Gallistel and colleagues for averaged vs. individual time courses of learning [37]). Indeed, inspection of the individual time courses (Fig 2B) indicates that some participants showed little color usage throughout all blocks of practice, while others departed from the 25% chance baseline at some point during practice. To follow up on whether group average increase in color usage was driven by the proportion of color users (rather than by the rate of color use increasing within individuals), we determined for each participant in each block whether the rate of color usage was *p* < .001 according to the binomial distribution by comparing the number of ambiguous trials with responses following color to the number to be expected by random responding. The chance of randomly responding in line with color (one out of four possible responses, 25% chance baseline) in more than nine of the 16 ambiguous trials in a block is *p* < .001. Thus, we were able to plot the percentage of participants showing significant color usage per block of practice (Fig 2C). The average rate of color usage (Fig 2A) and the proportion of color users (Fig 2C) both reached the asymptote at approximately the same time during practice (around Block 10). Apparently, increases in the average rate of color usage were generally brought about by participants who started to use color at different points in practice–rather than by continuous increases in color usage within (all) participants.

In the Appendix, we report on color usage separately for each color. With few exceptions, participants either reached color usage at the *p* < .001 level for all colors or for none. Most participants who eventually started to use color did so for all colors at about the same trial in the experiment.

#### Deviant test trials

Rate of color usage in the ambiguous trials might be considered a sensitive measure of the impact of the covarying feature (color) on response selection as the influence of the instructed feature (position) is minimal. However, in the last three blocks we pitted the instructed feature against the covarying feature. Test trials now consisted of stimuli that were fully spatially distinguishable, but color and corner were paired differently than on standard trials. In addition to the ambiguous-throughout and the ambiguous-after-learning condition, analyses included the data of the no-ambiguous group (participants who had never experienced ambiguous trials during learning, but experienced deviant trials in this later test phase). Again: If ambiguous trials during learning foster instead of merely measure color use, then color use and/or slowing by incongruent colors in position-color incongruent deviant trials should differ across the three groups.

**Fig 3:**
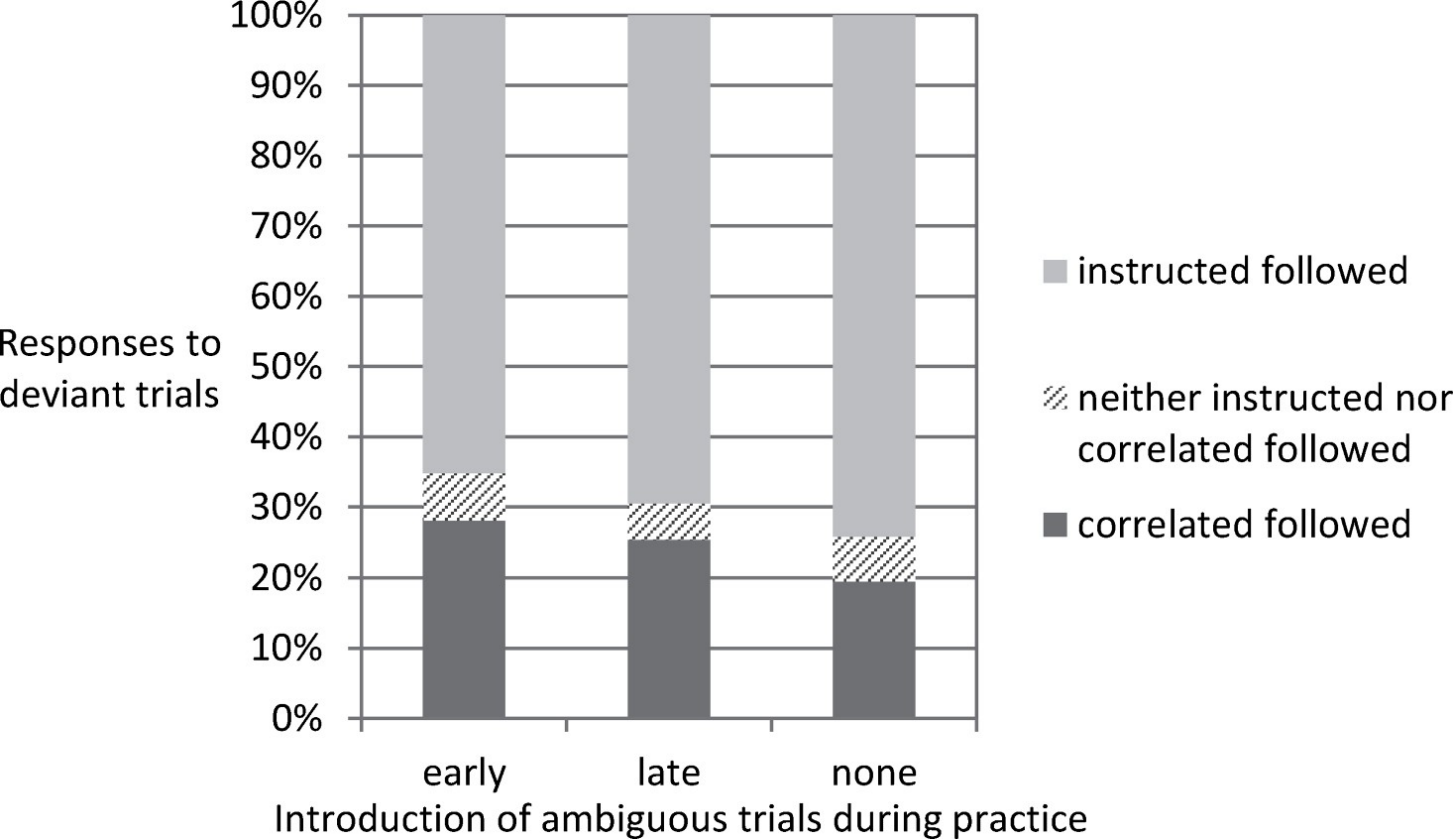
Exp. 1: Responses when pitting instruction against learning. Responses in deviant trials in the ambiguous-throughout condition, the ambiguous-after-learning condition, and the no-ambiguous condition. As the stimulus position is clearly distinguishable and the usual coupling between position and color is violated, this allows pitting response selection according to the instructed stimulus feature position against response selection according to color.

On average across the three groups (see Fig 3), participants responded in line with color rather than the instructed feature in 24.34% of the deviant trials. In 69.65% of the deviant trials they responded in line with stimulus position. Only 6.1% of the responses were errors that neither followed the instructed dimension nor the (formerly) covarying dimension. As in a setup with four response options there is only one option for making an error that is driven by the (former) color-position covariation, but there are two buttons that would count as a regular error, the latter rate can be divided by two for comparing the amount of either type of errors.

We tested whether the rate of the two variants of errors (color-based response vs. response neither in line with color nor with position) would differ from another and whether this was affected by group. The ANOVA of group and error type showed a main effect of error type, *F*(1, 57) = 36.77, *MSE* = 367.74, *p* < .001, *η*_*p*_^*2*^ = .392. There was neither a main effect of nor an interaction with group (*F*s < 1). Thus color-based responding in deviant trials was not affected by the amount of ambiguous trials presented before. Note that the same result would be obtained with the conservative approach (i.e., refraining from dividing the non-color errors by two). Additionally we checked whether participants would stop responding to color over the three blocks containing deviant trials. While there was a significant reduction from first (*M* = 27.92%) to last deviant block (*M* = 21.04%), *t*(59) = 2.16, *p* = .035, the average rate of color following in deviant trials in the last block was still substantially larger than the error rate in standard trials in the last block (*M* = 9.11%; *t*(59) = 3.29, *p* = .002).

**Fig 4:**
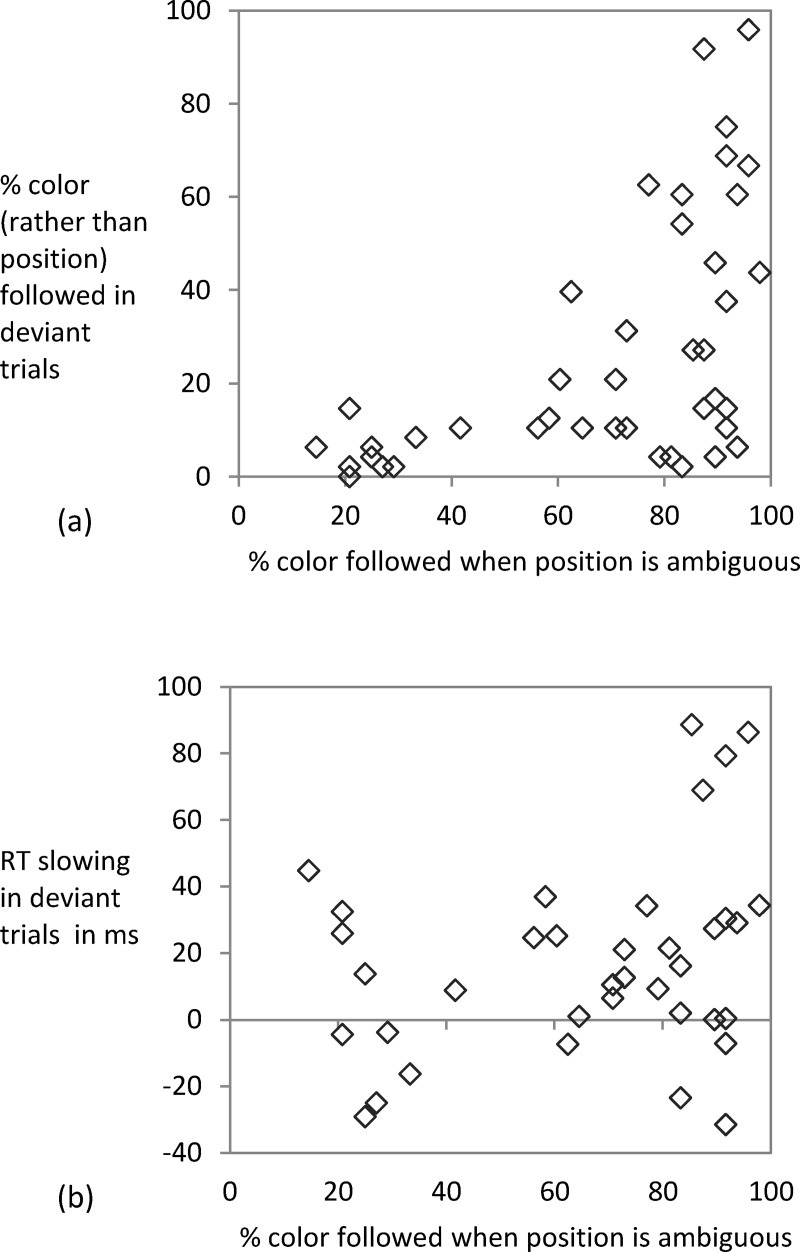
Exp. 1: Color usage in ambiguous vs. deviant trials. For the ambiguous-throughout condition and the ambiguous-after-learning condition the proportion of color usage in deviant trials is plotted against the proportion of color usage in ambiguous trials in Panel a. The RT slowing in deviant trials (standard trial RT subtracted from RT in deviant trials with response according to instruction) is shown in Panel b.

For the ambiguous-throughout condition and ambiguous-after-learning condition, we could compare color usage in deviant trials (*M* = 28.02% and 25.28%) and ambiguous trials (*M* = 64.27% and 72.82%, last three blocks). The mixed ANOVA of type of test trial and group only showed a main effect of type of test trial, *F*(1, 40) = 116.9, *MSE* = 314.57, *p* < .001, *η*_*p*_^*2*^ = .745 (other *F*s < 2.13). For all but two participants the rate of color usage was higher in ambiguous than in deviant trials (one showing an equal rate of 95.83%; the other person reaching 87.5% vs. 91.67%). While the average data suggests that the participants mostly relied on the instructed rather than the covarying feature when learning and instruction were pitted against each other, there was large interindividual variability. Fig 4A plots color usage in ambiguous trials (last three blocks) against color usage in deviant trials during the test phase (Kendall-Tau Correlation = .448, *p* < .001). While some participants strongly relied on the learned irrelevant feature even when it was put in conflict with the instructed feature, others used color only in the ambiguous trials–when the instructed feature could not be used to select a response. Usage in conflict trials paired with lack of usage in ambiguous trials was not observed.

#### Effects on RT

Even when participants responded as instructed on deviant trials, they might have been influenced by color. Indeed, RTs of correctly responded deviants were slower (*M* = 633.5ms) than RTs of standard trials (*M* = 604.96ms). The mixed ANOVA of experimental group (ambiguous-throughout, ambiguous-after-learning, no-ambiguous) and trial type (standard vs. deviant), showed a main effect of trial type, *F*(2, 57) = 17.29, *MSE* = 1403.77, *p* < .001, *η*_*p*_^*2*^ = .233. There was no main effect of group, *F*(2, 57) = 2.2, *MSE* = 28580.93, *p* = .12, *η*_*p*_^*2*^ = .072. Somewhat surprisingly, there was an interaction of group and trial type, *F*(2, 57) = 4.47, *MSE* = 1403.77, *p* = .016, *η*_*p*_^*2*^ = .135. Slowing in deviant trials with correct responses relative to standard trials was least pronounced for the ambiguous-throughout condition (*ΔM* = 10.21ms, *SD* = 25.26ms) and largest for the ambiguous-after-learning condition (*ΔM* = 56.23ms, *SD* = 66.84ms), with the no-ambiguous condition showing an intermediate amount of slowing (*ΔM* = 19.17ms, *SD* = 56.4ms).

Follow-up analyses suggested that there was no correlation between color usage in deviant trials and RT slowing in deviant trials that were responded according to the instructions (Kendall-Tau Correlation = .097, *p* = .283; across participants from all three groups). Thus, even participants who did not follow color in the deviant trials were slowed when the color-position correlation was violated. Apparently, some participants suppressed using color in deviant trials while using it in ambiguous trials. Yet, when suppression was not necessary, color usage and slowing were correlated: Color usage in ambiguous trials (last 6 blocks) was positively related to RT slowing in deviant trials (*r* = .303, *p* = .005; see Fig 4B).

Note that we additionally (see Appendix) analyzed the proportion of color-responses in ambiguous and deviant trials as well as correct responses in standard trials at each quintile of the response time distribution. This confirmed that our fast-paced covariation learning paradigm indeed led to color usage across the entire response time distribution. Alternatively, one could have suspected that color is used only in special trials. For instance, it would have been conceivable that color usage is reserved to very slow trials in which participants presumably ruminate on how to respond to a stimulus that cannot be discriminated on the instructed response feature or is shown only in fast deviant trials in which participants fail to control the influence of the covarying feature.

#### Explicit knowledge

In the post-experiment interview, 76% of the participants of the ambiguous-throughout condition (as protocols were not obtained in the other conditions) could perfectly report which color had been presented at which position. There was a high (Kendall-Tau) correlation between the number of correctly reported color-position relations and proportion of color usage in ambiguous (*r* = .592) and deviant trials (*r* = .599, *p*s = .004). There was perfect overlap between (a) *p* < .001—level color usage in the ambiguous trials of the second half of practice and (b) correctly reporting all 4 color-position pairings in the interview. All color users reported correctly, while none of the persons not using color did so.

### Discussion

In Experiment 1, we tracked how covariation learning leads to responses based on a non-instructed stimulus feature–despite that there was a perfectly valid instructed stimulus-response (S-R) rule and there were no salient error- or cognitive conflict signals that hindered instructed response selection. Even though we instructed participants to use an easily memorized, spatially compatible stimulus-response mapping (respond to the location of a stimulus), with practice, many participants started to use the correlated stimulus color for response selection. When the covariation between stimulus position and color was violated in deviant trials at the end of the experiment, some participants selected responses according to color rather than the instructed spatial stimulus-response mapping. This suggests that cognitive conflict remained in part undetected once it occurred after extensive practice. In addition, responses to deviants that were in line with the instructions were delayed relative to standard trials, suggesting that the color-position covariation influenced responding even when usage was suppressed.

We assessed covariation learning between position and color continuously throughout practice by inserting spatially ambiguous stimuli. In these trials the stimulus feature mentioned in the instruction could not be used for response selection. Thus, the covarying feature of color did not have to overcome a strong competitor to influence response selection. While ambiguous trials had been used in [19], the current experiment enables us to rule out reactive effects. Applying ambiguous test trials from Block 1 onwards vs. only in the second half of the experiment did not affect the amount of color-based responses in these trials. Also, prior exposure to ambiguous trials did not affect the rate of color usage when color was pitted against the instructed stimulus-response mapping in deviant trials at the end of the experiment.

Performance changes were in line with criteria of voluntary strategy change (see Introduction): Usage of the color-position covariation was linked to awareness about this regularity in the task material. Also, we observed that the group average rate of color usage in ambiguous trials was driven by the proportion of participants using color (rather than by a steady increase of color-usage within participants). Extended practice led some participants to select responses based on color while others continued to exclusively rely on the instructed stimulus feature when determining which response to give. Yet, slowed responses on deviant trials were found for them as well. This suggests that the more than two thousand trials of practice on the covariation led to covariation learning in all participants, while overt usage for response selection was only observed for participants who decided to use color. Another test of voluntary strategy change was applied in Experiment 2.

## Experiment 2

Experiment 1 tracked how the usage of a non-instructed stimulus feature developed across blocks of practice. The main goal of Experiment 2 was to manipulate the frequency of exposure to specific pairings between instructed and covarying stimulus feature. We hypothesized that color usage would generalize across frequent and infrequent color-position pairs.

We presented some color-position combinations more frequently than others. Across nine blocks of practice this lead to a difference in exposure of specific color-position combinations. As in Experiment 1, we used four colors and four stimulus positions in fixed pairs. Yet, this time we instructed a 4:2 mapping of stimulus positions to keys in order to be able to vary the frequency of color-positions pairings without at the same time varying the frequency of the responses. For instance, the left key was to be pressed for an upper right and a lower left stimulus, while the right key was due in case of an upper left or a lower right stimulus. Thus, each participant was instructed with two spatially compatible and two incompatible stimulus-response mappings. Previously [19] we used the same mapping, but that study lacked a frequency variation.

The experimental variation of color-position pairings was designed to allow an exploration of whether the spatial compatibility of the instructed stimulus-response mapping might affect the acquisition and usage of covariation knowledge about the non-instructed color stimulus feature. While Experiment 1 showed that cognitive conflict is not necessary for the usage of the non-instructed stimulus feature to occur, this does not rule out that frequent incompatible trials might be a motivating factor. It is conceivable that incompatible spatial mappings of the stimulus position and required response foster color usage. On the one hand, color usage might–on trial level–be stronger for colors usually paired with spatially incompatible instructed S-R mappings. On the other hand, it might be stronger for participants (across their color-position pairings) for whom the frequently presented position-color compounds are incompatible rather than compatible (see e.g., [34], for results suggesting that conflict frequency influences strategy change).

While some participants were more frequently exposed to incompatible color-position pairs (*incompatibles frequent condition*), with compatible pairs displayed infrequently, for other participants the setup was reversed (*compatibles frequent condition*). Thus, in both conditions there were frequent color-position pairs as well as infrequent pairs. For participants in the control condition, all color-position pairs occurred with the same frequency and the frequency of compatible and incompatible trials was balanced. We included the between-subjects factor of conflict frequency in order to explore whether cognitive conflict would foster color usage. In line with Fröber and Dreisbach as well as Dignath and colleagues, a higher proportion of incompatible trials might lead to changes in control settings [38, 39], such that participants more quickly and more strongly use the covarying feature of color rather than continuing to rely on the instructed feature. Furthermore, comparing performance on compatible and incompatible trials within subjects can help to assess whether participants can flexibly use the information on covariation in a trial-by-trial manner in situations where this strategy delivers the best results. On the one hand, Forrin and MacLeod [40] reported that contingency learning influences performance only when the nominally irrelevant feature is faster to process than the relevant feature. This might be the case on trials in which the instructed feature involves an incompatible mapping. On the other hand, Gaschler and colleagues reported that changes to an alternative strategy of information processing have been reported to affect either all or none of the stimuli [27]. In line with this, manipulating the ratio of stimuli not fitting the alternative strategy affected the rate of participants using the strategy for all stimuli [41] rather than leading to trial-by-trial variation in usage.

### Methods

#### Participants

We tested 85 participants (58 female, mean age 24.5 years, *SD* = 4.7 years, four left-handed). They participated in the study in return for course credit or €7 financial compensation. We randomly assigned 30 participants to the incompatibles frequent conditions and 31 to the compatibles frequent condition. In addition we tested the control condition (no frequency variation) with 24 participants. Given the higher guessing probability in the two-responses format compared to the four-responses format of Experiment 1, we increased sample size per between-subjects condition.

#### Task and procedure

Stimuli, task and procedure were similar to Experiment 1, with the following exceptions. Participants performed 10 blocks with 128 trials each. From Block 1 onwards, there was a fixed assignment of color to stimulus position (the specific color assigned to a position was balanced across participants). We did not start with random blocks as Experiment 1 had shown that onset of color usage usually took place after several blocks of practice and did not affect all participants. Thus, random blocks in the beginning were not needed as a baseline. Two of the colors were presented in 16 trials per block each. The other two colors were each presented in 48 trials per block (3:1 ratio of frequent to infrequent colors). Of these trials, we reserved four trials (3.1%) per color in each block as test trials (ambiguous trials in Blocks 1 to 9 and deviant trials in Block 10). As a result, each of the infrequent color-position combinations occurred in 12 trials per block (16 minus 4) and each of the frequent combinations in 44 trials per block (48 minus 4). Thus, participants needed more than three blocks to accumulate the same number of exposures of color-position combinations with the two infrequent colors that they accumulated in one block for the frequent pairs.

Participants responded with their left and right index fingers on the “4” and “6” keys on the number pad of a German keyboard, covered with a white circle. The 4:2 mapping made it necessary to differentiate between two variants of deviants: When the usual pairing between stimulus color and stimulus position was violated in Block 10, the color could either suggest the usual (learned) or a different response. In *response deviants*, the color did not match the usual pairing *and* suggested a different response than the instructed feature stimulus position. In *stimulus deviants* a different than the usual color was paired with a particular stimulus position, yet (based on the stimulus position it had been paired with) it signaled the same response as the instructed feature.

Half of the participants were instructed to press the left key in case the stimulus was closest to the upper right or lower left corner of the reference frame. Conversely, they were to press the right key in case the stimulus was closest to the upper left or lower right corner. Thus, the mapping was compatible for the lower stimulus positions (left = left key; right = right key) and incompatible for the upper stimulus positions. For the other half, the mapping was incompatible for the lower and compatible for the upper stimulus positons (press left for upper left or lower right position, press right for upper right and lower left position).

Orthogonal to the instructed mapping, for half of the participants the upper stimulus positions (and assigned colors) were used frequently and the lower ones infrequently. For the other half, the lower stimulus positions were frequent. Thus, half of the participants frequently experienced incompatible trials (while compatible trials were infrequent). For the other participants, the instructed S-R mapping only infrequently led to incompatible trials.

In summary, all participants experienced incompatible and compatible trials (according to the instructed stimulus-response mapping) and all participants experienced trials with frequent and with infrequent color-position combinations. Participants differed with respect to whether incompatible trials were frequent or infrequent. In the control condition (no frequency manipulation), the same mapping was used and the same number of trials was performed while using all color-position assignments with equal frequency.

### Results

#### Screening of the data

Data of 13 participants were excluded, because their error rate on standard trials exceeded 25% (eight in the incompatible frequent condition). One was excluded for reporting limited color vision, leaving us with 22 participants in the incompatible frequent condition, 28 in the compatible frequent condition and 22 in the control condition. We first analyzed how compatibility of the instructed stimulus-response mapping and color frequency affected how the *amount* of color usage in ambiguous trials developed with practice. Then we assessed the *onset* of color usage. Finally, we analyzed how compatibility of the instructed stimulus-response mapping affected responses when the instructed stimulus feature was pitted against the formerly covarying feature in deviant trials at the end of practice.

Before focusing on the influence of color-position pairings (i.e., our hypothesis on generalization across frequent and less frequent color-position pairings), we first tested whether response choice in ambiguous trials was influenced by compatibility of instructed S-R mappings. While the latter was not the case, we found evidence for generalization. Analyses of the deviants and standard trials documented that the compatibility manipulation was effective: Compatibility of the instructed S-R mapping affected performance unless position was ambiguous.

#### Frequency of incompatible trials

**Fig 5:**
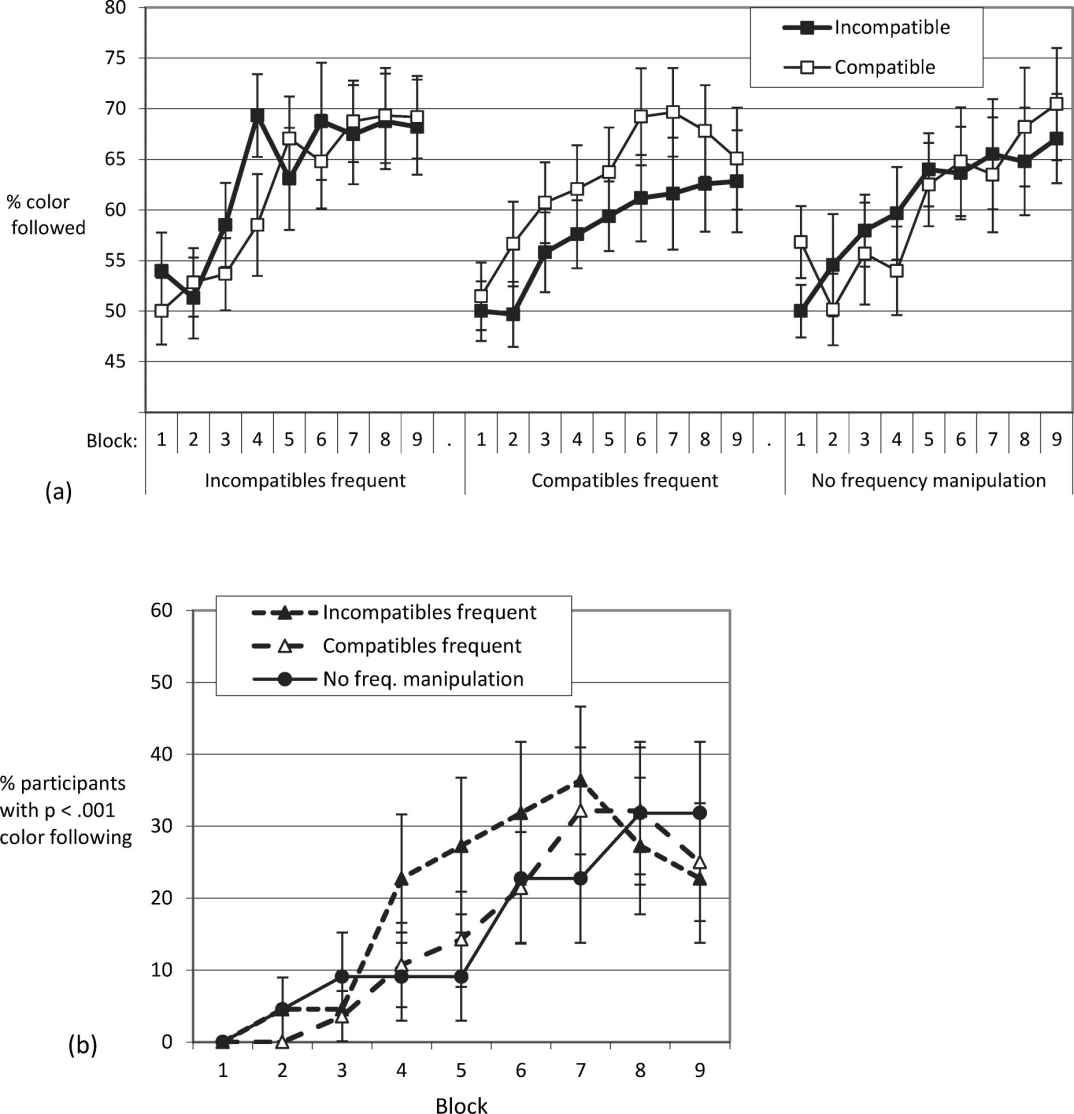
Exp. 2: Color usage in ambiguous trials. Panel (a) shows the average color usage in ambiguous trials increasing across blocks of practice for frequent and infrequent color-position pairings and for participants who experience spatially incompatible stimulus-response mappings frequently vs. infrequently. Panel (b) shows the practice-related increase in the proportion of participants who strongly use color. Error bars depict the between-subjects standard error of the mean.

Fig 5A shows how the rate of color usage in ambiguous trials increased with practice for the incompatibles frequent condition, compatibles frequent condition, and the control condition. We separately analyzed colors usually paired with a spatially compatible stimulus-response mapping and those with incompatible mappings. If, for instance, the upper left stimulus was blue for a participant and had to be responded to with the right key, the impact of this color-position pairing involving a spatially incompatible mapping would be assessed by presenting ambiguous blue stimuli. While the color array was presented centrally in the ambiguous stimulus, the color was associated with a spatially incompatible position. The mixed ANOVA including the three groups (incompatibles frequent, compatibles frequent, control condition) as a between subjects factor as well as block of practice (1 to 9) and compatibility (color which is paired with an incompatible vs. a compatible spatial position in standard trials) showed a main effect of practice, *F*(5.28, 364.64) = 11.35, *MSE* = 687.69, *p* < .001, *η*_*p*_^*2*^ = .141. There was no main effect of compatibility (*F* = 1.25). Yet, we obtained an interaction of compatibility and group, *F*(2, 69) = 4.74, *MSE* = 303.35, *p* = .012, *η*_*p*_^*2*^ = .121. This indicated that frequency of color-position pairs (rather than compatibility) was influencing color usage (other *F*s < 1). In the incompatibles frequent condition the incompatible colors (*M* = 63.26%) showed more color usage than the compatible ones (*M* = 61.56%). In the compatibles frequent condition the rate was higher for the compatible colors (*M* = 62.92%) than the incompatible colors (*M* = 57.84%). The control condition showed no difference between incompatible colors (*M* = 60.79%) and compatible ones (*M* = 60.67%). Thus, frequency of color-position pairing rather than compatibility of instructed mapping was driving color usage in ambiguous trials.

The lack of a main effect of group suggests that color usage was not robustly stronger for participants experiencing incompatible trials frequently. Neither was there an indication that the overall rate of color usage was influenced by the rate of incompatible trials, nor that participants flexibly used color on a trial-by-trial basis depending on whether the stimulus was compatible or incompatible (i.e., lack of a main effect of compatibility). While the results suggest that the spatial compatibility of the instructed stimulus feature did not influence the usage of the alternative feature of color in ambiguous trials, Fig 5A shows that color usage was higher for colors from more frequent color-position pairings.

In order to check robustness of the results to the decision of excluding participants with a high error rate (see above), we repeated this analysis with all participants included. The pattern of results was identical. The same main effects and interactions were found / not found either way.

#### Frequency of color-position pairings

Complementary to the compatibility analysis (see above), the data on the two conditions with a variation of frequency of color-position pairs (i.e., incompatibles frequent and compatibles frequent groups) allow to test for a within-subjects effect of frequency of color-position pairing on the rate of color usage in ambiguous trials. The mixed ANOVA including block of practice as well as the frequency of color-position pairing as within-subjects factors and group (incompatibles frequent vs. compatibles frequent) as a between-subjects factor again showed a main effect of practice, *F*(5.14, 247.17) = 7.82, *MSE* = 751.53, *p* < .001, *η*_*p*_^*2*^ = .14. There was a main effect of frequency of color-position pairing, *F*(1, 48) = 7.98, *MSE* = 318.38, *p* = .007, *η*_*p*_^*2*^ = .143, (other *F*s < 1.99). The rate of color usage was higher for the frequent, as compared to the infrequent pairings. Again, the pattern of results was the same when including the participants with high error rate.

As in Experiment 1, practice-related changes in the average rate of color usage were driven by changes in the proportion of participants showing significant color usage in a block (Fig 5B). The two-choice format employed in Experiment 2 implied that >13 of 16 ambiguous trials in a block had to be responded in line with color for crossing the *p* < .001 criterion in the binomial test. The less sensitive measure (as compared to the four choice setup used in Experiment 1), in addition to the high error rate due to spatially incompatible trials might explain the overall low rate of strong color users and the lack of a robust difference between conditions.

#### Increase of color usage per exposure to color-position combination

**Fig 6:**
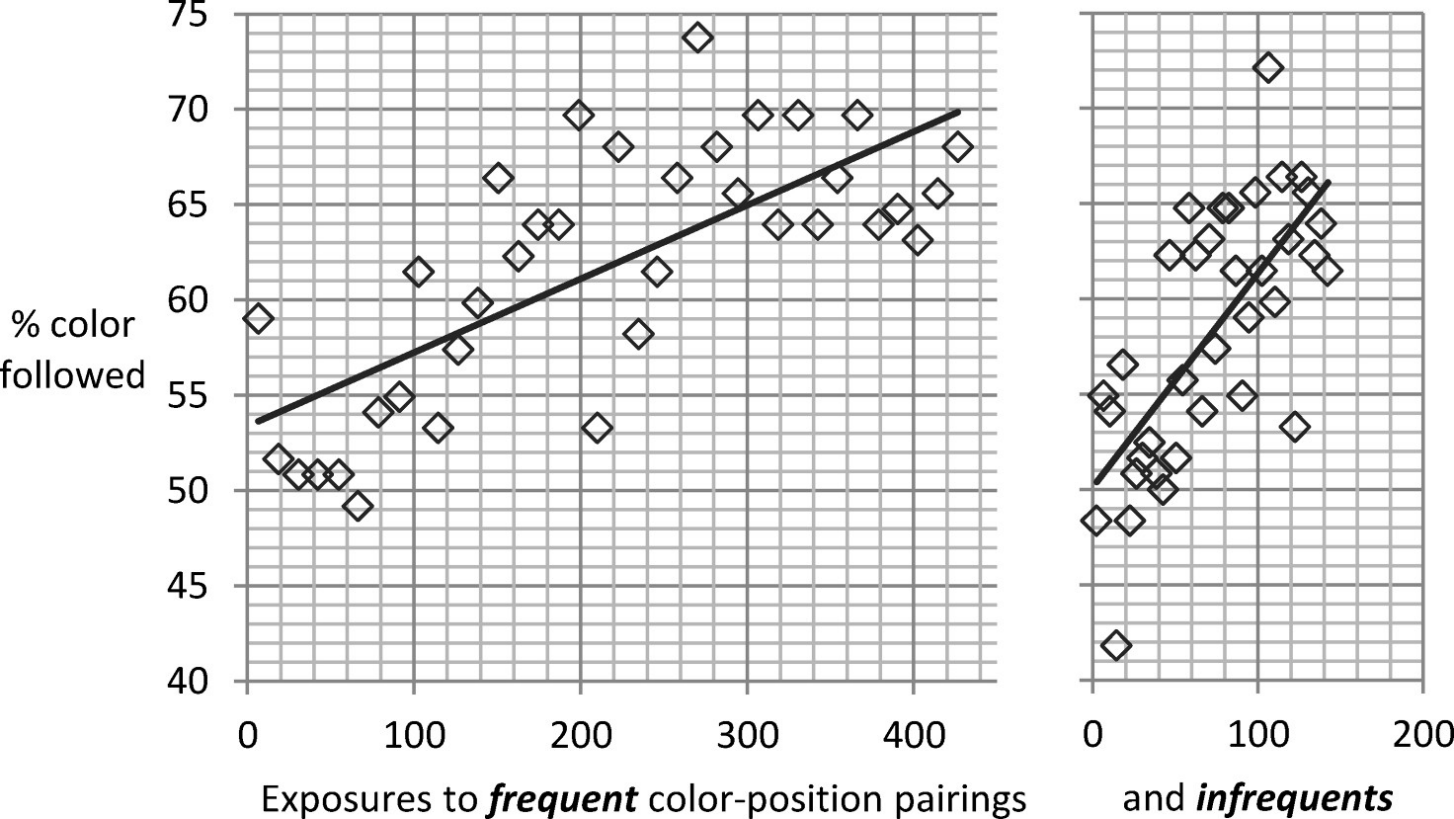
Exp. 2: Color usage per exposure to color. The increase in % color usage in ambiguous trials (y-axis) in relation to the amount of exposures to frequent (left part of figure) and infrequent (right part of figure) color-position combinations.

The above analyses show that the rate of color usage increased with practice and was higher for colors in frequent color-position pairings than for infrequent colors. Yet, in order to analyze whether color usage might generalize from frequent to infrequent color-position pairings, the increase in color usage has to be examined in relation to the number of exposures to specific color-position pairings. Over the course of nine blocks of practice, each of the two frequent colors was shown 432 times and each infrequent color was shown 144 times. If color usage generalizes from frequent to infrequent color-position pairs, the increase in color usage per exposure should be steeper for infrequent as compared to frequent colors. Fig 6 suggests that this was indeed the case. To compare at what rate color usage increased per exposure to a frequent vs. infrequent color-position pair, we calculated the linear slope of the number of exposures to the color on the percentage of color usage for each participant of the two conditions with frequency variation. This slope was steeper for infrequent colors (*M* = .132% increase per exposure) than for frequent colors (*M* = .04% increase per exposure; *t*(49) = 3.66, *p* < .001).

Inspection of Fig 6 might suggest that for the frequent colors there is an asymptote in color following the last third of the experiment. This raises the question whether the rate of color usage was increasing differently even in the first half of the experiment. Similar to the analysis based on the whole experiment, this post-hoc analysis yielded *M* = .175% increase per exposure for infrequent and a rate of *M* = .068% for frequent colors, *t*(49) = 1.89, *p* = .032, one-tailed.

#### Onset of color usage for frequent and infrequent colors

Apart from the effects of the frequency of color-position pairs on the *amount* of color usage, we analyzed the impact of color-general vs. specific exposure to the covariation on the *onset* of color usage. This allowed for another test of whether color usage generalizes from frequent to infrequent color-position pairs. We used the CUSUM method to determine the trial in which a participant started to use a specific color (cf. Durstewitz et al. [42]; for an adaptation to a task similar to the current one see [19]). This method uses the cumulative sum of the difference between the average performance (per participant and color) and current performance (at the specific point in practice) to calculate the point in the time series where the upward change relative to the overall mean level sets in. The trial of onset of color usage was determined individually for each color in each participant. The analysis is only meaningful for participants who eventually start to use color. There were 20 participants from the frequency variation groups (10 of each) who showed a number of color-following responses in ambiguous trials which was *p* < .001 according to the binomial distribution (> 89 of the 144 ambiguous trials; as there were only two response keys, chance baseline was 50% in Experiment 2).

**Fig 7:**
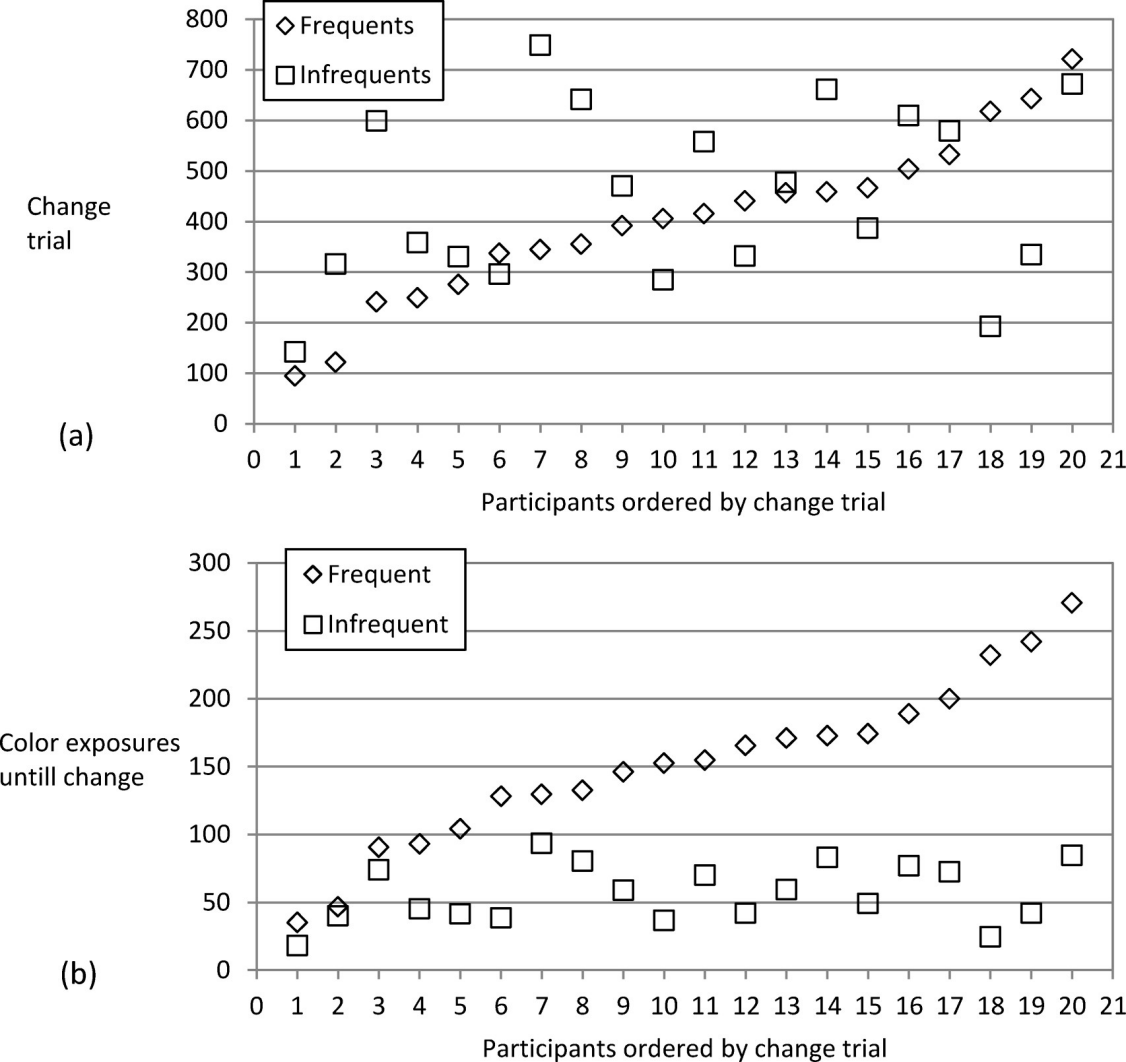
Exp. 2: Onset of color usage. Panel a shows the trial (y-axis) at which (according to the CUSUM method) a participant starts to use a colors for response selection in ambiguous trials separately for frequent and infrequent colors. Participants (x-axis) are sorted based on color usage in frequent colors. Panel b shows how many exposures to a frequent or infrequent color-position combination had passed at the point the onset of color usage was located.

We analyzed onset of color usage from two perspectives that differed in how practice was measured. One can either ask at which *trial* in the experiment color usage started for a frequently or infrequently shown color (Fig 7A). Alternatively, one can ask after how many *exposures* to the specific color-position pair color usage started for a specific color (Fig 7B). The *trial* of the onset of color usage did not differ for colors from frequent (*M* = 402.7 trials) and infrequent color-position pairs (*M* = 449.4 trials; *t*(19) = 1.03, *p* = .316). This implied that a strong difference was to be obtained when testing after how many *exposures* to the specific color-position pair the onset of color usage happened. Participants needed to accumulate *fewer* exposures to infrequent color-position pairs (*M* = 56.5 exposures) than to frequent color-position pairs (*M* = 151.4; *t*(19) = 7.28, *p* < .001) before the onset of color usage. Taken together, the results on the increase of color usage per exposure (section above) and the data on the onset of color usage suggest that color usage generalizes across frequent and infrequent color-position pairs.

#### Deviant test trials

**Fig 8:**
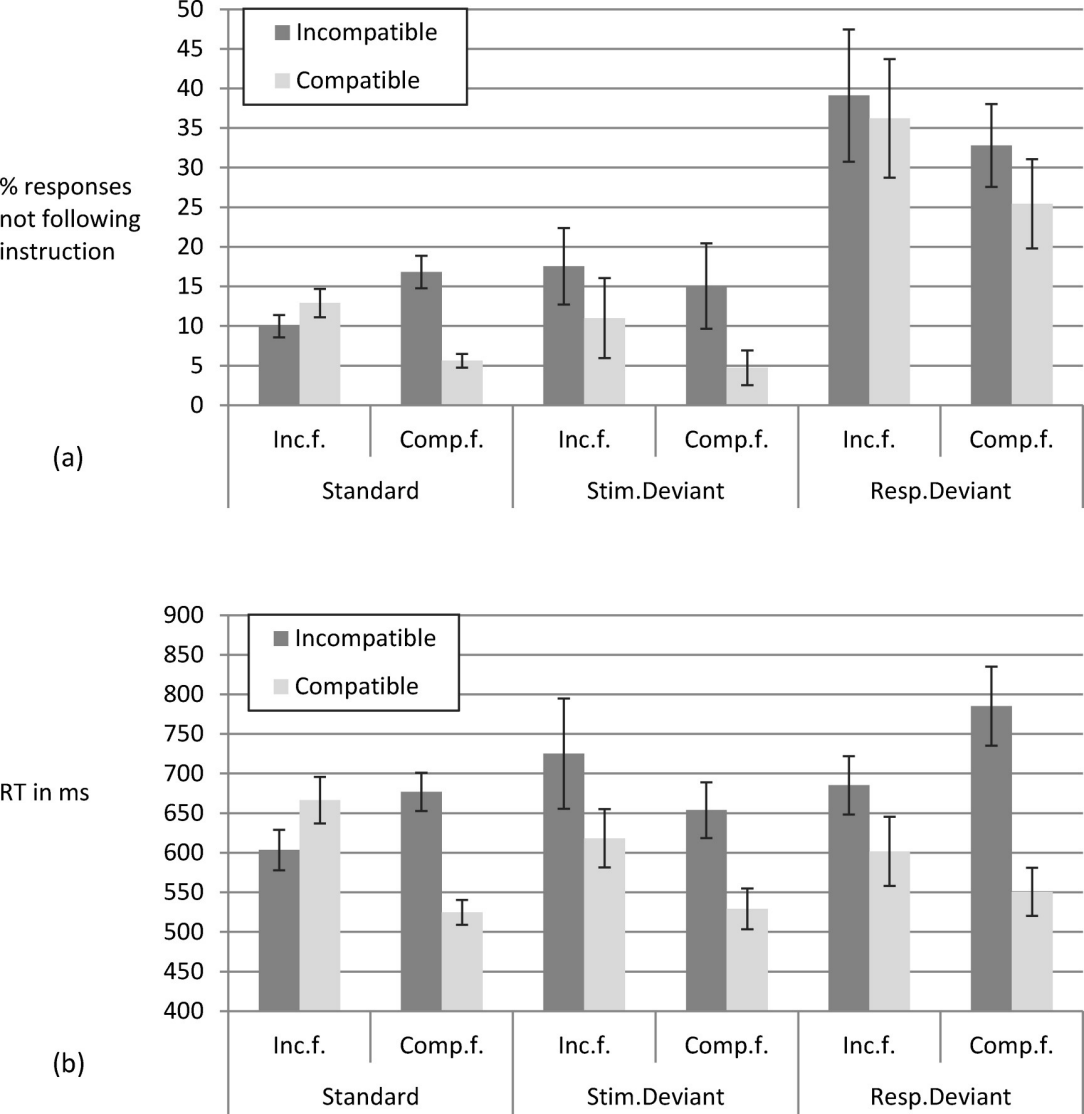
Exp. 2: Spatial compatibility. Panel (a) shows the % of responses not following the instructed stimulus feature position for standard trials, stimulus deviant trials, and response deviant trials of Block 10, differentiating between spatially compatible and incompatible trials for the compatibles frequent and the incompatibles frequent condition. Panel (b) shows mean RTs. Error bars depict the between subjects standard error of the mean.

Analyses of the deviant trials aimed at testing whether color would influence response selection and/or speed of response selection when pitted against the instructed stimulus feature position. Furthermore, this allowed a more sensitive test of the potential effects of compatibility of the instructed S-R mapping, as compatibility was present in the displayed stimulus position (rather than merely as an association of color and usual position, as in the ambiguous trials). This could rule out the possibility of unsuccessful manipulation of compatibility. Conceivably, participants could have, for instance, re-coded the stimulus positions mapped to the responses as positive and negative diagonal. We report only the results from the two frequency variation conditions, as, due to a programming error, the control group participants did not receive deviant trials. As depicted in Fig 8A, the rate of not following the instructed stimulus feature in the *response deviants* (*M* = 33.38%) was higher than the error rate in standard trials in Block 10 (*M* = 11.33%) and in *stimulus deviants* (*M* = 12.08%).

The mixed ANOVA, including group (incompatibles frequent vs. compatibles frequent) as the between-subjects factor and within-subjects factors trial type (stimulus deviant vs. response deviant) and compatibility (incompatible vs. compatible spatial stimulus-response mapping), showed a main effect of trial type, *F*(1, 48) = 18.62, *MSE* = 1200.46, *p* < .001, *η*_*p*_^*2*^ = .28, and a main effect of compatibility, *F*(1, 48) = 6.61, *MSE* = 343.82, *p* = .013, *η*_*p*_^*2*^ = .121, other *F*s < 1.59. The rate of off-instructions responses was higher in response deviants than in stimulus deviants and higher for incompatible rather than compatible trials. As Experiment 2 used only two responses, not following the instructed stimulus feature in response deviants could either be based on using color or on committing an error. Yet, the higher rate of off-instructions responses in response deviants compared to stimulus deviants suggests that participants indeed used color instead of the instructed feature position.

Repeating the above ANOVA with RT in correct deviant trials as dependent measure also showed a main effect of compatibility, *F*(1, 48) = 32.61, *MSE* = 28527.89, *p* < .001, *η*_*p*_^*2*^ = .405. In addition, there was an interaction of trial type and group, *F*(1, 48) = 5.57, *MSE* = 24295.96, *p* = .022, *η*_*p*_^*2*^ = .104, as response deviants were by *ΔM* = 76.56ms slower than stimulus deviants in the compatibles frequent condition, while they were by *ΔM* = 28.22ms faster than stimulus deviants in the incompatibles frequent condition. There was a tendency of an interaction of compatibility and group, *F*(1, 48) = 3.09, *MSE* = 28527.89, *p* = .085, *η*_*p*_^*2*^ = .06, as the slowing by incompatible as compared to compatible trials was in tendency larger in the compatibles frequent condition (*ΔM* = 179.69ms) than in the incompatibles frequent condition (*ΔM* = 95.13ms), other *F*s < 1.47. Both interaction effects are in line with the proportion congruent effect (i.e., larger congruency effect if there are many rather than few congruent trials, cf. Logan and Zbrodoff, [43]).

**Fig 9:**
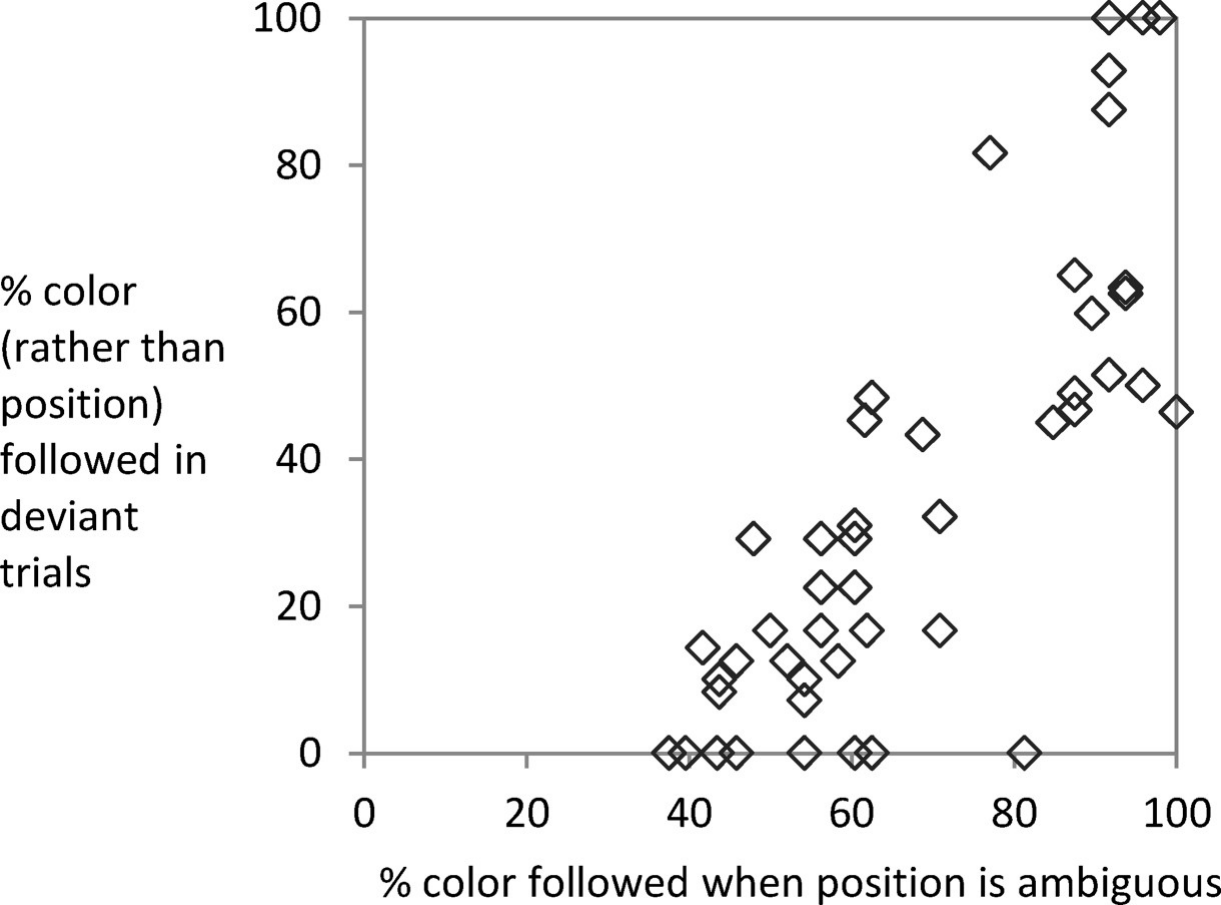
Exp. 2: Color usage in ambiguous vs. deviant trials. The proportion of color usage in ambiguous trials (in last three blocks; x-axis) is plotted against the proportion of color usage in response deviants (y-axis).

The proportion of response deviants responded to according to color rather than position was higher for participants who showed a high rate of color usage in ambiguous trials (see Fig 9, Kendall-Tau Correlation = .618, *p* < .001; color usage in last three blocks of ambiguous trials). As in Experiment 1, there were some participants showing a high rate of color usage both when the instructed feature could not be discriminated and when it was pitted against the correlated feature. Yet, in contrast to Experiment 1, there were no participants showing strong color usage in ambiguous trials only. As in Experiment 1, there was no correlation between color usage in deviant trials and RT slowing in deviant trials that were responded according to the instructions (Kendall-Tau Correlation = .042, *p* = .674, for response deviant trials). RT slowing in stimulus deviant trials did not correlate with the proportion of trials responded to according to the instructions either (*p*s > .6 for the correlation with color following in response-deviant and stimulus-deviant trials). Differing from Experiment 1, color usage in the ambiguous trials (last three blocks) did not correlate with deviant slowing either (*r* = .121, *p* = .221).

#### Compatibility and frequency affecting RT and error rate in standard trials

Error rates and RTs in standard trials of Block 10 reflected that performance was affected both by the spatial compatibility of the instructed response feature as well as by the frequency of the color-position pair. While error rate and RT (Fig 8) were higher in incompatible than compatible trials for participants of the compatibles frequent condition, the compatibility effect was reversed (and smaller) for participants of the incompatibles frequent condition. For them, the incompatible trials were less error-prone and faster than the compatible trials. Yet, these trials featured frequent color-position pairings. Thus, the impact of color frequency in part overrode the compatibility effect.

For error rate, the mixed ANOVA including group (incompatibles frequent vs. compatibles frequent) as between-subjects factor and the within-subjects factor compatibility (incompatible vs. compatible spatial stimulus-response mapping), showed a main effect of compatibility, *F*(1, 48) = 11.36, *MSE* = 37.05, *p* = .001, *η*_*p*_^*2*^ = .191, and an interaction of compatibility and group, *F*(1, 48) = 33.21, *MSE* = 37.05, *p* < .001, *η*_*p*_^*2*^ = .409 (*F* < 1 for the main effect of group). Similarly, applying the same ANOVA to RT showed a main effect of compatibility, *F*(1, 48) = 6.31, *MSE* = 7764.2, *p* = .015, *η*_*p*_^*2*^ = .116, and an interaction of compatibility and group, *F*(1, 48) = 36.68, *MSE* = 7764.2, *p* < .001, *η*_*p*_^*2*^ = .433 (*F* = 1.45 for the main effect of group).

#### Explicit knowledge

All four color-position pairings were reported correctly by 63.6% of the participants of the incompatibles frequent condition, and by 67.9% of the participants of the compatibles frequent condition. In the control condition, 59.1% of the participants could correctly report all four pairings. The number of pairings reported correctly was positively correlated with the proportion of color-based responses in ambiguous trials (*r* = .404, *p* = .02; *r* = .499, *p* = .001; *r* = .45, *p* = .01; Kendall-Tau correlations for participants of the incompatibles frequent, compatibles frequent and control condition). The correlation with proportion of color responses in response deviants were positive but not significant (*r*s > = .226).

### Discussion

According to the theories that base strategy change on an interplay of learning about the regularities in the task material plus a decision to use this knowledge, strategy change can generalize from frequently encountered to infrequent stimuli. Thus, performance for infrequent stimuli can change faster than for frequent stimuli, if practice is counted in terms of number of exposures to the specific stimuli (see Gaschler et al. for an elaboration on this point [27]). Yet, frequent and infrequent stimuli should show strategy change at the same rate per exposure to specific stimuli according to the theories that base strategy change exclusively on learning about specific instances of stimuli as in the instance theory by Logan [5].

Our results suggest that participants generalized knowledge about the covariation across frequent and infrequent color-position pairs. The proportion of color-based responses per block of practice was higher for ambiguous trials with colors from frequent rather than infrequent color-position pairs. This suggests that strategy change started with the frequent stimuli. Yet, the increase in color usage per exposure to a color-position combination was steeper for infrequent as compared to frequent color-position pairs. This suggests generalization. Another indicator of generalization of color usage from frequent to infrequent colors was obtained when analyzing after how many trials vs. after how many exposures to a specific color-position pair the onset of color usage was located. The onset of color usage occurred at about the same trial in the experiment–hence fewer exposures to infrequent than frequent color-position pairings were necessary for the onset of color usage to occur.

Spatial compatibility of instructed stimulus-response mappings did not influence color usage in ambiguous trials. However, RT and error rates in standard trials showed that spatial stimulus-response compatibility of the instructed feature influenced performance together with the covarying feature of color. This finding is relevant as it shows that spatial compatibility of the instructed mappings was indeed effective. Alternatively, it could be suspected that participants could have re-coded instructions in terms of diagonals (positive diagonal = left hand, negative diagonal = right hand). The lack of an impact of compatibility on color usage in ambiguous trials therefore cannot be explained by doubting that spatial compatibility was successfully manipulated at all. While we did not find a dosage effect for the amount of incompatible trials, there might have been a general effect. In contrast to Experiment 1 (no incompatible trials), we did not observe cases of participants using color only in the ambiguous trials (when the instructed feature could not be used) while foregoing to use color in deviant trials (when the instructed feature could be discriminated). Rather, incompatible trials in Experiment 2 might have led participants with sufficient covariation knowledge to apply it for strategy change. Potentially, participants received sufficiently many incompatible trials, leading those who had gained explicit knowledge about the task regularity to use this knowledge for strategy change (cf. Touron and Hertzog [25, 26]). Future studies should apply further variations of dosage to test for a between-person effect of the number of incompatible trials. The lack of a within-person effect of compatibility on color usage in ambiguous trials (lack of difference in performance in colors associated with an incompatible vs. compatible position) is in line with our earlier work on strategy change [34, 41] where participants either applied the alternative strategy to all stimuli or none–rather than applying it in a trial-by-trial manner to the stimuli where it might be most useful.

The 4:2 mapping furthermore allowed an examination of whether color-usage was (exclusively) driven by learning the covariation between stimulus color and response position or, alternatively, also by learning the covariation between stimulus position and stimulus color. Deviant trials in which the stimulus color did not match the learned stimulus position, but nevertheless matched the response usually paired with this color (i.e., stimulus deviants) were slowed and more error prone. This suggests that covariation learning involving the two stimulus features contributed to performance.

## General discussion

Many studies have pointed out that instructions can determine the stimulus features by which responses are being selected (cf. the study [44] and the review [45] by Meiran and colleagues), as long as they can be effectively represented (cf. Duncan et al. [46]). While studies by Braem and colleagues as well as by Wenke et al. focused on how practice can help in fine-tuning instructed task sets [47, 48], the current study asked whether and how practice could lead to a spontaneous strategy shift based on an (apparently) irrelevant stimulus feature, resulting in response selection based on the non-instructed covarying stimulus feature rather than exclusive reliance on the instructed stimulus feature. Taken together, the results suggest that spontaneous strategy change was linked to awareness and under voluntary control: While extended practice led some participants to base response selection on color, others continued to exclusively determine responses based on the instructed stimulus feature. Those shifting to a color-based strategy in part seemed to have stopped checking the instructed stimulus feature. They continued using color for response selection even when the covariation was violated. Apparently for them cognitive conflict remained undetected once it occurred. In addition, strategy change generalized to infrequently practiced stimuli.

In Experiment 1, we instructed a spatially compatible mapping of stimulus position to responses and observed that many participants with practice started to use the covarying feature of color for selecting responses in ambiguous trials in which the instructed stimulus feature was not informative. Some even relied on color instead of the instructed feature when the color-position covariation was violated in deviant trials. Given the work on conditional and unconditional automaticity in spatial tasks by De Jong et al [49], one might argue that we have selected a hard test for a non-instructed to-be-learned feature to compete against the instructed feature in response selection. Furthermore, participants were to pick up on the covariation of four colors and positions, while the Schuck et al. setup [19] with two colors and two responses presumably made it easier to learn about and use color.

We varied how early in practice ambiguous trials were inserted to measure usage of the covarying stimulus feature for response selection. The results ruled out that ambiguous trials produce the very phenomenon they are meant to measure. Hence, this technique might prove useful for many research questions involving covariation learning as it avoids problems of RT measures. For instance, for tracking the acquisition of color-word associations, Schmidt and De Houwer [50] have dealt with potential confounds of reaction time differences between frequent and infrequent pairings by general practice effects (i.e., early in practice participants are slow, so the same amount of extra slowing on a low frequency stimulus relative to a high frequency stimulus might indicate less knowledge in an early block as compared to a later block). Rather than using warm-up trials to reduce the confounding of RT by general practice effects, response choice in ambiguous trials might be used as an alternative.

In Experiment 1, the departure from instruction-based task processing was accompanied by awareness of the regularity in the task material. Earlier work has documented that strategy change involving skipping to process parts of stimuli that were task-relevant according to the instructions is accompanied by awareness (cf. Haider and colleagues as well as Gaschler et al. [21, 27]). Our results suggest that, incorporation of an alternative feature into the task set also seems to be accompanied by awareness. On a theoretical level this suggests that overcoming of task shielding (cf. Dreisbach and Haider [13, 14]) might lead to a cognitive conflict (instructed direction of attention vs. learned direction of attention) which might lead to awareness (cf. Haider and Frensch [51]). On a practical level the coupling with awareness suggests that participants can (at least prior to massive practice, see Cleeremans ans Jiménez, [52]) control usage of the learned feature. Likely they notice when they start using the learned instead of the instructed stimulus feature so control demands (cf. Gaschler et al. [27]) and self-control strategies might impact whether or not the strategy change takes place. In safety-relevant tasks on the job it might be necessary to avoid using a near perfect correlation between an instructed feature that is difficult to attend (e.g., train signal in a bend or at adverse light conditions) and a readily available correlated feature (e.g., state of the last signal, nodding of a colleague). Correlated features might drive expectations about features instructed as task relevant. For instance, Lawton and Ward [53] have documented that high-speed train drivers need to keep expectations active about when and where to decode which specific signal in the focus of attention. Under adverse light conditions or in bends, an eyeblink alone would be sufficient to miss the status of an important signal. In an analysis of over 100 Australian railway accidents, Edkins and Pollock [54] identified that expectation of a green signal was a common cause for drivers going through a red signal.

Voluntary control about using color for selecting responses might be the basis of the finding that some participants selected responses based on color only in the ambiguous trials, but followed instructions in the deviant trials (despite being slowed). Some of them continued to do so even when this was no longer suitable in the last blocks of the experiment. In our study, the participants applying the shortcut in ambiguous trials were aware of the color-position covariation. The results thus differ from unconscious shortcuts being wrongly applied when they no longer fit (cf. Woltz et al. [18]). Rather, some participants might have failed to notice that the shortcut no longer applies, while other participants became aware of the cognitive conflict between the instruction-based and covariation learning-based response selection. The latter might have performed the task like those being instructed to suppress what they have incidentally learned in a choice reaction task with regularities in the task material in the process dissociation procedure (cf. Destrebecqz and Cleeremans, [55]). Suppressing the usage of knowledge in such setups has been interpreted as an indicator of voluntary control in early studies by Ach and more recently by Jacoby [56, 57]. In line with this interpretation, varying the frequency of specific color-position compounds in Experiment 2 yielded further evidence for the involvement of a voluntary strategy change. While color usage was stronger for frequently as compared to infrequently presented color-position compounds, the difference between these two conditions was much weaker than what would be predicted by theories that base strategy change exclusively on the accumulation of specific instances in memory [5]. Rather, we observed generalization across frequently and infrequently practiced color-position pairs–based on rate of performance change per exposure as well as time-point of onset of color usage. Thus, participants needed fewer exposures to infrequent color-position pairings than frequent ones for starting to use color for response selection. We assume that this pattern reflects a two-stage process of incidental covariation learning plus voluntary strategy change which has been shown to affect highly practiced and less practiced or unpracticed stimuli alike by Haider and Frensch as well as by Gaschler and colleagues [28, 34].

To our knowledge, the generalization of covariation learning driven performance change has not been tested in other setups with a non-instructed but covarying stimulus feature. In word-color contingency learning [40, 50, 58], participants respond to the print color of (unrelated) words. RTs are lower for frequent color-word pairs as compared to infrequent ones. In the correlated flanker task [59–61], participants are to respond to a central target stimulus (i.e., letter or other symbol) and ignore the flankers. While the flankers never occur as targets and there is no instructed S-R mapping for the flankers, they covary with the target and the response. Given that covariation learning impacts performance despite that the flankers and the covariation are neither subject to awareness (cf. Miller, [60]) nor attention (cf. Mordkoff & Halterman, [61]), generalizations across frequently and infrequently presented pairings seem unlikely. Furthermore, while Miller [60] documented covariation learning between the flanker and the required response (rather than between flanker and target), the slowing in stimulus deviants in our Experiment 2 suggests that the associations between the instructed and the non-instructed stimulus feature were acquired and influenced performance. Furthermore, in word-color contingency learning and in the correlated flanker task, RT measures have documented that covariation learning can influence the *speed* with which participants respond–yet they mostly continue to respond in line with the instructed S-R mapping. We used ambiguous trials to track how covariation learning could impact *which response* is being selected. Future studies have to detail why our task might have led to awareness and voluntary strategy change. Unlike in other covariation learning tasks, we did not instruct participants to ignore the irrelevant stimulus feature. Color was just not mentioned. In contrast to the flanker task, spatial attention could not differentiate between the instructed vs. the alternative stimulus feature. The non-instructed feature was a feature of the object of which participants were to determine the location. This might have forced them to pay some attention to color, which might have been a necessary pre-condition for generating awareness and strategy change (cf. Rünger and Frensch [35] as well as Hoffmann and Sebald [62]). Future work should test whether other correlated features (e.g., shape) lead to similar results and determine how different factors influence the rate and extent to which a correlated stimulus feature is acquired and used. Potentially, the correlated feature has to be a feature of the attended object or graphically linked to it by a line as suggested by Baker and colleagues [63]. Conceivably, decreasing the covariation might reduce the acquisition of the feature, while even very few deviant trials in the learning blocks might block that covariation knowledge is used for selecting responses [34, 41]. Future research might also test interindividual difference factors that may lead to differential reliance on (usually valid) shortcuts. While for some characteristics (such as conscientiousness) the prediction might seem obvious, this is not the case for other variables such as working memory capacity. For instance, work by Duncan and colleagues [46] suggests that high working memory capacity should be linked to high quality implementation of the instructed task set. This might lead to strong reliance on the instructed task set. At the same time, distinct representations of instructed stimulus features and the mapped responses might be a pre-requisite for covariation learning to occur.

Interesting to explore in future work, learning-based strategy change might make use of covariation knowledge with respect to delays and by this employ non-instructed features that can be more general than specific perceptual characteristics of stimuli or action effects (cf. Aufschnaiter et al. [64] for covariation learning of task and delay in task-switching). Delays of action effects often covary with outcomes and demands for corrections in everyday human-computer interaction (cf. Thomaschke and colleagues, [65, 66]). For instance, an unexpected long waiting time for the sound of the paper tray suggests the printer has a problem.

Given that in the correlated flanker task, participants do not, with practice, start to pay attention to the non-instructed stimulus feature and do not become aware of it, it seems plausible that their representation of the task does not change. Likely, they keep performing a react-to-the-central-stimulus task, relying on the instructed S-R mapping. Thus, covariation learning changes performance while the instructed task set remains intact and dominates despite practice on stimulus-response pairings (cf. Dreisbach and Haider, [13, 14]). Covariation learning thus leads to changes in performance *within* the instructed task. For the correlated flanker task [60], Miller discussed (and ruled out) the option that performance changes due to covariation learning were accompanied by awareness, a shift of attention (to the irrelevant feature) and a change in task representation. Yet, there are reports from research with other paradigms that seem to be in line with this possibility: For instance, Tran and Pashler [67] suggested that a covariation of an irrelevant and a relevant stimulus feature in a video game had a strong impact on performance in participants who had become aware of the predictive relationship and engaged in overt anticipation of a non-instructed cue. Presumably, for them the cue had become part of the task set. Similarly, in our experiments, the instructed spatial task might have changed into a space-and-color task–at least for the participants using the non-instructed feature of color in the ambiguous trials, but refraining from color-based response selection in deviant trials. For participants who had based responses on color even when pitted against the instructed feature, the instructed task set might have become considerably weakened or replaced. In a similar vein, Schuck et al. [19] had not only found that color became represented in prefrontal areas, but also reported that this was accompanied by a decrease in the representation of the instructed stimulus feature position.

Qualitative changes in task performance have been reported in work with the Nissen and Bullemer Serial Reaction Task (SRT, [68]) in case of simple (i.e., first order) sequential regularities [20, 35, 69], or instructions telling participants about the sequential structure (cf. Tubau et al. [70]). In the latter study, participants spontaneously changed from reacting to stimuli (in line with the choice reaction task instructions) to producing a sequence from memory. Thus, while the task was instructed as a choice reaction task, with practice it became a memory-based production task. While the SRT has been useful for studying the conditions under which incidental learning leads to a strategy change from choice reaction to sequence production [35], the current task allows the tracking of whether–and how–a non-instructed covarying stimulus feature gains influence over response selection.

**Appendix**

**Generalization across specific colors in Experiment 1**

**Consistency in the usage of color**

We observed an all-or-none pattern with respect to whether a color was used at all. For participants in the ambiguous-throughout condition and participants in the ambiguous-after-learning condition we determined for each color whether color usage was *p* < .001 in the ambiguous trials of the last nine blocks of practice (i.e., the blocks in which both conditions had ambiguous trials). Significant usage of all four colors was observed for 69.05% of the 42 participants while 16.67% did not show significant color usage for any of the four colors. Thus, for 85.71% of the participants there was full consistency: Either all colors were used or none was used in ambiguous trials at *p* < .001. Conversely, inconsistent color usage was obtained for only six (14.29%) participants. Four of them reached a significant level in only one of four colors, while two crossed the *p* < .001 threshold with three of four colors. A two vs. two color case was not observed.

**How quickly the participants started using color information**

To further characterize how learning leads to the usage of a non-instructed stimulus feature for response selection, we explored *when* participants started to use specific colors. It is conceivable that participants learned about the relation of one or two colors to the correct response early on and used this knowledge for response selection in ambiguous trials while other colors were learned late or never. We used the CUSUM method to determine the trial in which a participant started to use a specific color (cf. [42]; for an adaptation to a task similar to the current one see [19]). This method uses the cumulative sum of the difference between average performance (per participant and color) and present performance (at the specific point in practice) to calculate the point in the time series where the upward change relative to the overall mean level sets in. The trial of onset of color usage was determined individually for each color in each participant. The analysis is only meaningful for participants who eventually start to use color. We thus determined the change points per color for the 14 (out of 20) participants of the ambiguous-throughout condition who showed a *p* < .001 rate of color usage according to the binomial distribution.

S1 Fig suggests that some participants started to use color early on in practice and others started later. Visual inspection suggests that the onset occurred consistently across the four colors and stimulus positions for seven participants. S1 Fig shows for each participant who eventually used color according to the binomial test criterion (sorted on the x-axis) at which trial in practice (y-axis) color-usage started for a specific color. In order to quantify the extent of consistency, we calculated the within person standard deviation of onset trials across the four colors (see Touron [71]). A small standard deviation means that the trials of onset of color usage closely correspond for a given participant. Given that only some of the trials are ambiguous trials (which we used for testing color usage), they can’t fall on the same trial. Thus, some variation must even be shown by participants who start with all colors at once. The average within-person standard deviation of onset trials of the 14 participants of the ambiguous-throughout condition was 191.41 trials (range 40.04 trials to 483.66 trials of the 2688 trials in the learning phase).

We used a randomization test (cf. Good [72]) to be able to evaluate this level of consistency. The randomization test uses the observed distribution of shift points. This avoids investing into assumptions on either a fixed shift probability per time point or a specific time course of shift probabilities. In the randomization test we randomly sorted the onset trials for each color across the 14 participants. By this, the onset trial for green of one participant was paired with the onset trial for blue of another participant, etc. This gave us 14 standard deviations across the four onset trials based on randomly shuffling the colors across persons. We used 10,000 repetitions of this random shuffling and obtained an average pseudo within standard deviation of 382.82 trials. Obtaining a larger variability in random shuffling than in assessing the variability of onset of color usage underlines the impression from S1 Fig: Participants differed with respect to when in the learning phase they started to use color while at least some participants showed a once-for-all colors onset. More importantly, none of the 10,000 random simulations yielded a lower standard deviation than obtained in the sample. Thus, the probability of obtaining the level of within-person consistency as high or higher based on random variation is *p* < .001. Despite that only some participants clearly showed a once-for-all colors onset of color usage, the average level of consistency was robustly distinguishable from chance. The results are in line with the inference that color usage generalized across colors.

**Quintile analyses for Experiment 1**

Short presentation times were meant to enforce fast responding, instead of rumination–in case of ambiguous stimuli. We determined the proportion of color usage in ambiguous and in deviant trials (and the proportion of correct responses in standard trials) for each reaction time quintile (determined per participant and trial type). For ambiguous trials, these quintile analyses (S2 Fig) suggested that the rate of color usage was similar across the reaction time distribution, *F*(4, 160) = 2.29, *MSE* = 209.45, *p* = .062, *η*_*p*_^*2*^ = .054 (taking into account the ambiguous trials in the last three blocks). Note that the effect of RT quintile on proportion of color usage did not differ for the ambiguous early vs. ambiguous late group, nor was there an interaction of quintile and group (*F*s < 1.04). The homogenous rate of color usage across the RT distribution suggests that people did not only rely on the covarying feature of color when they were processing the stimulus slowly, but also did so when responding fast. As depicted in S2 Fig, this was the case despite the finding that the reaction time distributions of ambiguous trials, deviant trials and standard trials taken from the last 6 Blocks of the Experiment differed substantially in the tail of the distribution. While similar speed was reached in the fastest quintile (430ms, 449ms and 444ms, for standard, ambiguous and deviant trials), responses of the slowest quintile of ambiguous trials were by 227ms slower than the slowest responses to standard trials (while deviant trials were slowed by only 86ms in the slowest quintile).

In deviant trials color usage varied systematically across the RT distribution, with higher rates in the fastest and the slowest quintile. There was a main effect of quintile, *F*(2.61, 104.54) = 6.94, *MSE* = 180.93, *p* = .001, *η*_*p*_^*2*^ = .148; Greenhouse-Geisser corrected). Again, there was no main effect of, nor an interaction (*F*s < 1) with the group factor (ambiguous early vs. late).

## Supporting information

S1 Fig Exp. 1: Change trials per color. The trial (y-axis) at which (according to the CUSUM method) a participant starts to use colors (or a color) for response selection in ambiguous trials is depicted across colors (C1 to C4) and participants (x-axis, participants sorted). Large interindividual variability with respect to how early color usage sets in is paired with high intraindividual consistency across color-position pairs.
(TIF)

S2 Fig Exp. 1: Responses per RT quintile. The graph plots the percentage of correct (or color-followed) responses per quintile of the RT distribution for standard, ambiguous and deviant trials.
(TIF)
