# Supplementary figures and images for: Incidental covariation learning leading to strategy change
Source: PLoS One. 2019 Jan 24;14(1):e0210597. doi: 10.1371/journal.pone.0210597 (PMC6345462; doi:10.1371/journal.pone.0210597)

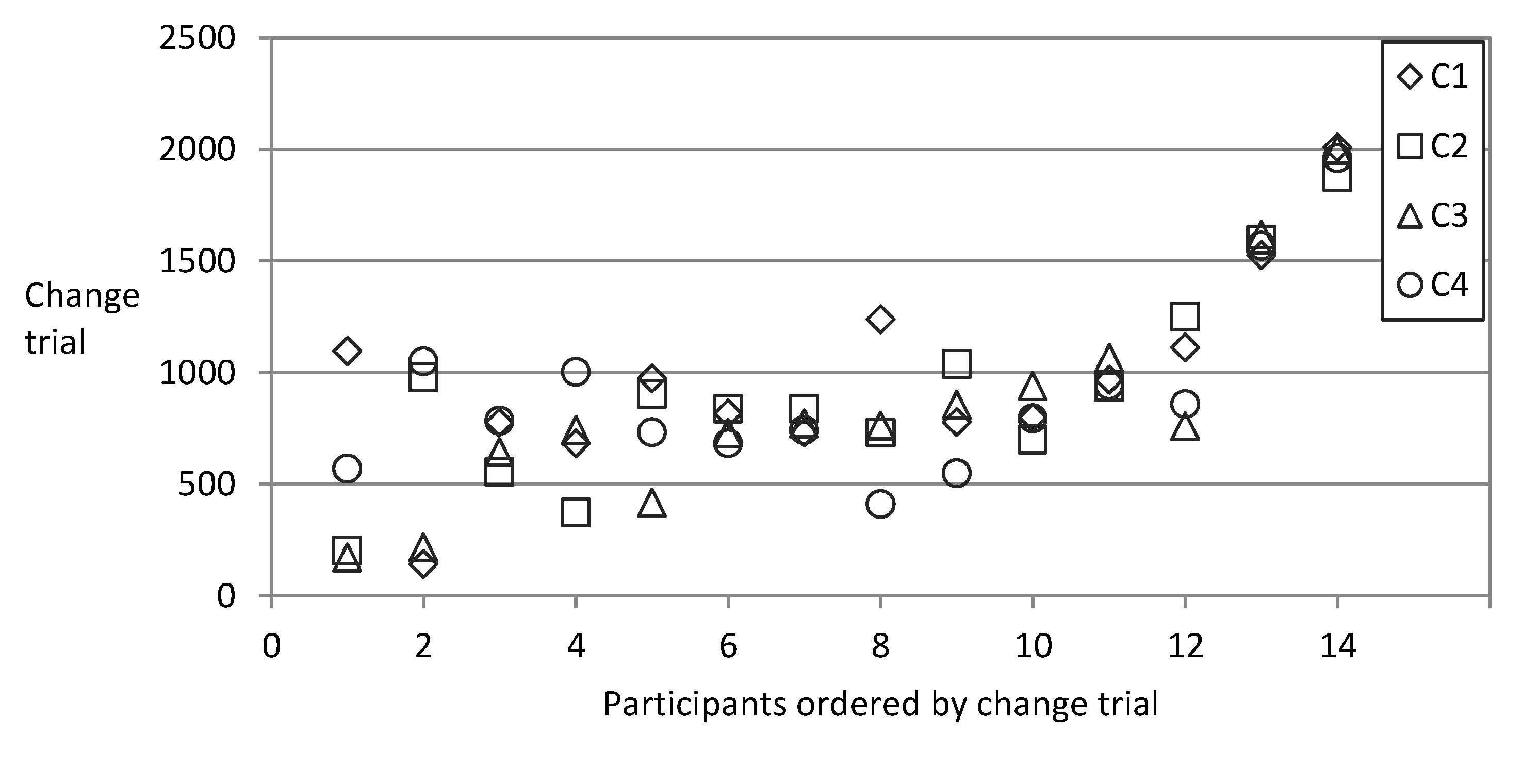

Supplement: S1 Fig — The trial (y-axis) at which (according to the CUSUM method) a participant starts to use colors (or a color) for response selection in ambiguous trials is depicted across colors (C1 to C4) and participants (x-axis, participants sorted). Large interindividual variability with respect to how early color usage sets in is paired with high intraindividual consistency across color-position pairs. (TIF) [file pone.0210597.s001.tif]

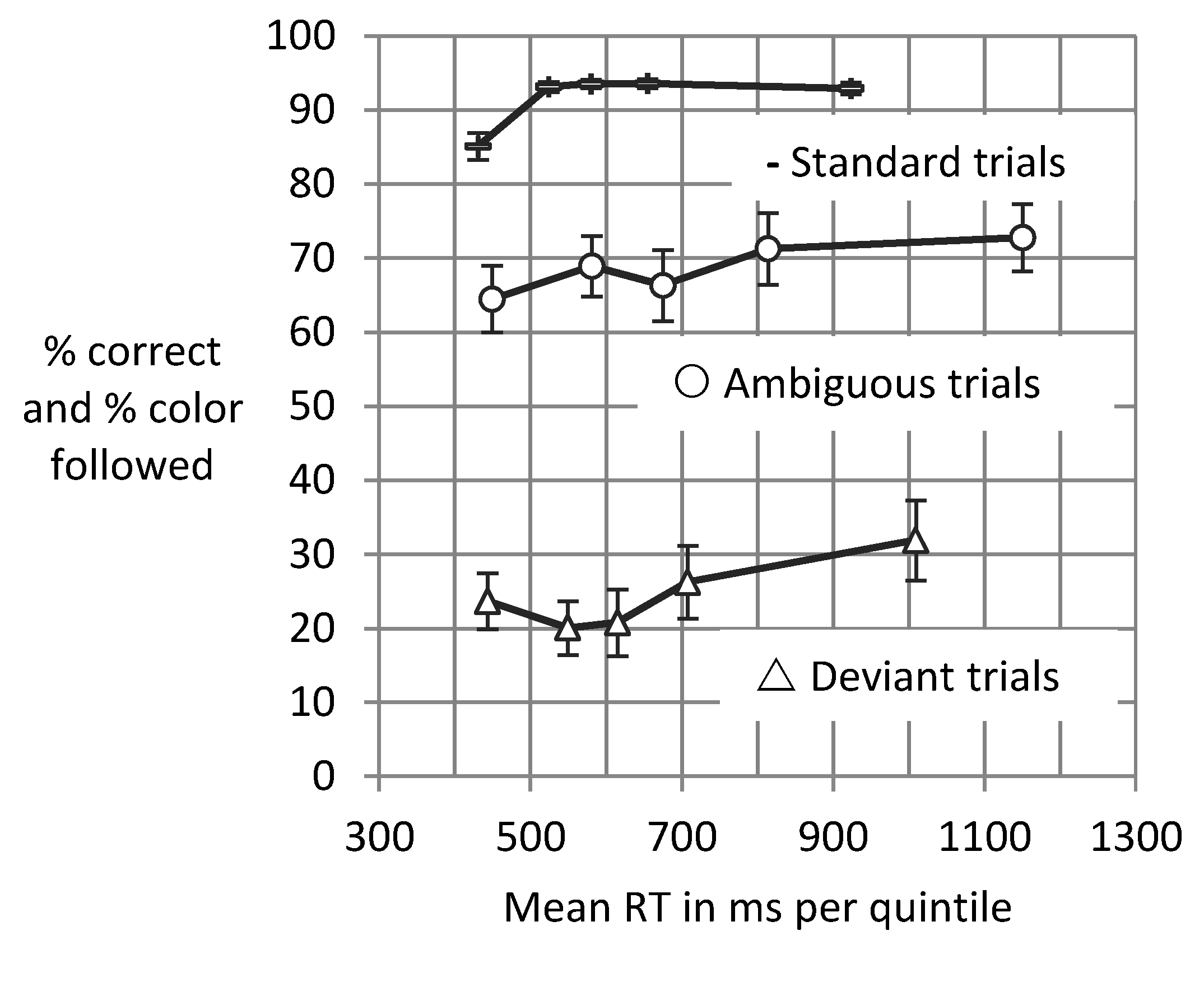

Supplement: S2 Fig — The graph plots the percentage of correct (or color-followed) responses per quintile of the RT distribution for standard, ambiguous and deviant trials. (TIF) [file pone.0210597.s002.tif]
